# Legume-supplemented feed for children hospitalised with severe malnutrition: A Phase II trial

**DOI:** 10.1017/S0007114524000837

**Published:** 2024-06-04

**Authors:** Kevin Walsh, Agklinta Kiosia, Peter Olupot-Oupot, Florence Alaroker, William Okiror, Margaret Nakuya, Tonny Ssenyondo, Denis Amorut, Charles Bernard Okalebo, Rita Muhindo, Ayub Mpoya, Elizabeth C George, Gary Frost, Kathryn Maitland

**Affiliations:** 1 Division of Diabetes, Endocrinology and Metabolism, Imperial College, 6th Floor Commonwealth Building, Hammersmith Campus, DuCane Road, London W12, UK; 2 Dept. of Nutritional Sciences. School of Life Course & Population Sciences, Faculty of Life Sciences & Medicine, King’s College, London SE1 9NH, UK; 3 Mbale Clinical Research Institute, Busitema University Faculty of Health Sciences, Mbale Campus, Palissa Road, PO Box 1966, Mbale, Uganda; 4 Soroti Regional Referral Hospital, Hospital Road, PO Box 289, Soroti, Uganda; 5 Kenya Medical Research Institute (KEMRI)-Wellcome Trust Research Programme, Kilifi, Kenya; 6 Medical Research Council Clinical Trials Unit (MRC CTU) at University College London, UK; 7 Imperial College, Department of Infectious Disease and Institute of Global Health and Innovation, Faculty of Medicine, Imperial College, London, UK

**Keywords:** Severe malnutrition, Clinical Trial, Legume-based feeds, African children

## Abstract

Children hospitalised with severe malnutrition have high mortality and readmission rates post-discharge. Current milk-based formulations target restoring ponderal growth but not modification of gut barrier integrety or microbiome which increase risk of gram-negative sepsis and poor outcomes. We propose that legume-based feeds rich in fermentable carbohydrates will promote better gut health and improve overall outcomes.

We conducted an open-label Phase II trial at Mbale and Soroti Regional Referral Hospitals, Uganda involving 160 children aged 6 months-5 years with severe malnutrition (mid-upper arm circumference (MUAC) <11.5cm and/or nutritional oedema). Children were randomised to a lactose-free, chickpea-enriched legume paste feed (LF) (n=80) versus WHO standard F75/F100 feeds (n=80). Co-primary outcomes were change in MUAC and mortality to Day 90. Secondary outcomes included weight gain (>5 g/kg/day), *de novo* development of diarrhoea, time to diarrhoea and oedema resolution.

Day 90 MUAC increase was marginally lower in LF versus WHO arm (1.1 cm (IQR 1.1) vs 1.4cm (IQR 1.40) p=0.09; Day 90 mortality was similar 11/80 (13.8%) vs 12/80 (15%) respectively OR 0.91 (95% CI 0.40 -2.07) p=0.83. There were no differences in any of the other secondary outcomes. Owing to initial poor palatability of the legume feed 10 children switched to WHO feeds. Per protocol analysis indicated a trend to lower Day 90 mortality and readmission rates in the legume feed (6/60: (10%) and (2/60: 3%) vs WHO feeds (12/71: 17.5%) and (4/71: 6%) respectively.

Further refinement of legume feeds and clinical trials are warrented given the poor outcomes in children with severe malnutrition.

## Introduction

Severe malnutrition remains a frequent cause of hospitalisation in African children. It is associated with high in-hospital mortality rates of ~20%^(1; 2)^ and poor long-term outcomes^(3; 4)^. Clinical trials addressing infection prophylaxis^(3)^ or modification of the standard recommended World Health Organization (WHO) feed^(5)^ have failed to improve the poor outcomes. Milk-based feeds recommended for management of severe malnutrition (called F75 and F100) result in nutritional (anthropometric) recovery in survivors (the current gold standard of success). Nevertheless, this poorly predicts short and long term outcomes^(6)^, including increased risk of life-threatening events (death and/or re-hospitalisation with pneumonia or diarrhoea) in the 12 months following initial admission^(4; 7)^. A Phase II trial examining other formulations compared a feed with reduced lactose and carbohydrate load in the starter feed compared to standard formula (F75). This did not demonstrate improvement in outcomes indicating more radical approaches are required in the design of nutritional feeds^(5)^.

There are multiple lines of evidence indicating that several domains of gut function are aberrant in children with severe malnutrition. Intestinal atrophy^(8; 9)^ results in functional loss of brush border disaccharidases (lactase, maltase and sucrase)^(10; 11)^ which exacerbates diarrhoea and impairs recovery. Moreover, there is a significant relative microbiota immaturity and high levels of pathogenic flora in children with severe malnutrition which are only partially ameliorated following three weeks of standard nutritional interventions^(12)^. We hypothesized that intestinal mucosal integrity and gut microbial diversity can be restored in severe malnutrition by providing substrates that and induce fermentation in the gastrointestinal tract^(13)^. Fermentable carbohydrates can improve the balance of normal gut microbes and positively influence the immunological and metabolic function of the gut ^(14; 15)^. Carbohydrates that escape digestion in the gastrointestinal tract (resistant starch and dietary fibre) induce favourable changes in colonic microbiota fermentation ^(16)^. These lead to the generation of short-chain fatty acids (SCFA) which have a positive influence on gut integrity and nutritional health by improving energy yield, modulation of colonic pH, production of vitamins and the stimulation of gut homeostasis, including anti-pathogen activities ^(17; 18)^. We tested this hypothesis in a pilot trial (Modifying Intestinal Microbiome by Legume-Based fEeds: MIMBLE 1 PACTR201805003381361) which compared cowpea-supplemented standard nutritional formulae to standard WHO formuala (F75/F100)^(19)^. We demonstrated the feed was safe, palatable and resulted in equivalent weight and MUAC gain compared to standard WHO formulae (F75/F100)^(19)^. In the standard WHO feed arm faecal microbiota diversity showed very little change over the 28-day intervention nor change in major phyla. Furthermore, the SCFA concentrations on admission were approximately a third of the concentration of those reported in healthy African infants ^(20)^. However, in standard WHO feed arm, but not the cowpea arm there was a suppression of the SCFA propionate and butyrate at day 7 (to about 1/10^th^ of the normal concentrations) a period when the children are at high risk of mortality. We suspect that the suppression of SCFA at day 7 may have been due to the use of antibiotics, which recovered once antibiotic treatments were stopped. In-vitro batch culture (in an artificial colon) of the WHO milk feed (F75/100) demonstrated no impact on the gut microbiome or microbiologcal diversity whereas in the cowpea-enhanced feeds lead to increases in bifidobacteria (that has been linked to improved epithelial integrity^(21)^) and diversity. Since there were no differences on diarrhoea (frequency and resolution) or other clinical endpoints between the feeds, we made further modifications to the feed and developed, with a UK food manufacturer, a lactose-free, fermentable carbohydrate-containing alternative feed (Mimble 2 feed)^(22)^ swapping cowpea for chickpea. Here we report the Phase II Clinical Trial which compared a chickpea-supplemented lactose-free feed to standard (WHO) milk feeds on a range of endpoints. The trial was registered with **ISRCTN 10309022** on 23/05/2018.

## Methods

Between 5^th^ July 2018 and 28^th^ August 2019 we conducted an open-label, proof-of-principle randomised controlled trial on the paediatric wards in two sites (Mbale and Soroti Regional Referral Hospitals) in Eastern Uganda. The trial was designed to evaluate safety and efficacy of lactose-free chickpea based nutritional formulae compared to standard milk-based feeds recommended by WHO (control).

### Screening, Randomisation and blinding

Children with suspected severe malnutrition were clinically assessed for eligibility and exclusion criteria. Children aged 6 months to 5 years hospitalised with severe malnutrition were eligible for trial enrolment. Severe malnutrition was defined as either marasmus (defined by mid-upper arm circumference (MUAC) <11.5cm) ‘and/or’ kwashiorkor (defined as symmetrical pitting oedema involving at least the feet irrespective of weight for height Z score (WHZ) or MUAC). In children with life threatening complications, where prior written consent from parents/legal guardians could not be obtained, ethics committees approved parental verbal assent and deferred written informed consent as soon as practicable^(23)^. Otherwise, informed written consent was obtained from parents or guardians before randomisation. Children with severe malnutrition with a comorbidity at very high risk of death e.g. malignant disease or terminal illness, or a parent/guardian not willing to consent were not eligible for this trial.

An independent data manager, based at KEMRI-Wellcome Trust Research Programme (KWTRP), Kenya generated the sequential randomisation list, using permuted blocks. This sequence was used, by a study administrator at KWTRP, Kilifi, Kenya, to prepare randomisation cards with the treatment allocation which were sequentially numbered and sealed in opaque envelopes, each signed across the seal ensuring allocation concealment. In the hospitals in Uganda randomisation was done by the study clinician using the numbered envelopes sequentially which contained the randomised feed strategy. Nurses/doctors were unblinded to study intervention; laboratory tests were assayed blinded. Children were randomly assigned 1:1 to either legume-based paste feed (investigational) or F75/F100 feeds (control) recommended by WHO.

### Study interventions

The details of the content, development and nutrient profiles of the legume-based feed have been previously reported^(22)^ Briefly, the ingredients lactose-free skimmed milk powder (7.25%), rapeseed oil (11.5%), gram flour (10%), sugar (9%) and water. All ingredients were sourced from established EU suppliers and passed all required safety tests for human consumption as appropriate for each ingredient (contaminants, pesticides, toxins, bacterial contamination etc.). The final feed contains (per 100 ml): 200 kcal, 18 g total carbohydrate, 5.6 g protein, 12g fat, and 0.4 g resistant starch. The final product matched the macronutrient profile of double-concentrated F100 adhered to all relevant legislation regulating infant foods. Chickpeas were selected as the source of resistant starch, since they are widely grown and eaten throughout Africa. Micronutrient (vitamins and minerals) content could not be matched in this ready-to-use product, so this was replaced at the point of feeding. The detailed feeding protocol for legume-based is detailed in the published protocol)^(24)^ Children in the control arm received F75 and F100 as per WHO recommendations.

### Study procedures

Children were managed on general pediatric ward. A structured clinical record documented relevant clinical, examination and laboratory baseline assessment. Nutritional feeds were given per the published protocol^(24)^. Briefly, for those in the control arm (WHO feeds) initially 130 ml/kg/day F75 therapeutic milk was given at 4-hourly intervals over the day until the child was stabilized and demonstrating appetite. At this point they transitioned to 4-hourly F100 therapeutic milk at the same rate and increased by 10 ml per feed, until a maximum rate of 200 ml/kg/day was achieved. Legume feeds were provided as a paste 4-hourly at 45-50g/kg/day (or 35-40g/kg/day if oedematous). With additional water per feed provided starting at 105-110ml/kg/day. Feed weight and additional water volume was adjusted daily in accordance to increasing weight (using a feed volume/weight calculation chart). Once clinically stable the feed weight increased by 5g/feed until a maximum of 100g/kg/day. Mineral mix was added to the water for children in legume arm (as WHO F100 and F75 formulae already contained mineral mix). Thus, the quantity of legume feed provided matched the total amounts of energy and protein that would be received in the control arm, and additional water provided to match the fluid received. If the child took less than 80% of feed volume/weight for two consecutive feeds, despite attempts with spoon or syringe, then children were offered nasogastric tube feeding. Children in the legume strategy who could not initially tolerate non-fluid diet were switched to the WHO standard F75 feed and could then return to the legume strategy when non-liquid feeds were tolerated. All feed volumes and problems with feeding were recorded on standard proforma. Other standard treatments were prescribed including anti-malarials and antibiotics, following national guidelines.

Children were reviewed twice daily to discharge (generally ~day 14). On consenting for the MIMBLE 2 study patients/parents/guardians agreed to remain in hospital for a minimum of 7 days but preferably 14 days (based on both the WHO and Ugandan Ministry of Health guidelines). Patients were permitted to leave earlier if they had no oedema if applicable, good weight gain and MUAC > 12.5cm. Treatment with control/experimental treatment will be for 14 days duration, followed by standard treatment outpatient as required. This includes provision of ready to use therapeutic feeds until child has recovered, defined by a weight-for-height z-score >−2. At discharge children were back to the community nutrition programmes where were reviewed as per standard country guidelines. Children were reviewed by the study teams for clinical status and anthropometric status at 28- and 90-days post-randomisation. Serious adverse events (SAE) were actively solicited which included prolongation of hospital admission, readmission, mortality and suspected allergic reaction to the feed.

### Endpoints

The co-primary endpoint was change in mid-upper arm circumference (MUAC) at Day 90 and mortality at Day 90. Secondary outcome measures included change of weight and achieving a weight gain of >5g/kg/day by day 28 and day 90, parent-reported denovo development of diarrhoea (> 3 loose stools/day) and time to resolution of diarrhoea, time to oedema resolution and presence of oedema at days 28 and 90; and the number of serious adverse events (prolongation of hospital admission, readmission, mortality and suspected allergic reaction to the feed to Day 90).

### Ethics Statement

This study was conducted according to the guidelines laid down in the Declaration of Helsinki and all procedures involving human subjects/patients were approved by the Imperial College Ethics Committee, London, UK (Protocol number **17IC4146)** on 29^th^ May 2018 and Mbale Hospital Research Ethics Committee, Uganda (Protocol number **019/2018**) on 15^th^ March 2018. Written informed consent was obtained from the parents of all participants.

### Statistical Anaysis

Clinical data were analysed by using IBM Statistical Package for Social Sciences (SPSS) for Windows version 28 (Armonk, NY: IBM Corp) and R and R studio Core Team (2022). Calculation of weight for height (WHZ) was performed by using the online WHO Anthro Survey Analyser. Primary and secondary outcomes were analysed on an intention-to-treat and per-protocol basis. Per protocol analysis was defined as the analysis that included children who received and successfully completed their allocated treatment upon randomisation, during their hospital stay, and their survival status was known until study discharge (Day 90).

Differences in feed provision, anthropometric and clinical characteristics between the two groups on different follow-up days, were evaluated using the non-parametric Mann-Whitney U test due to the non-normal distribution of anthropometric data. For categorical data, such as diarrhoea and oedema status at baseline and on days 7, 28, and 90, chi-square analysis was employed. Cox regression analysis and Kaplan-Meier survival curves were employed to assess mortality and recovery differences between treatments, with competing risks regression analysis for readmissions, employing the Fine and Grey competing risk regression model, with the competing risk of death. Children were censored if they absconded (left hospital or nutritrional follow up against medical advice) or lost to follow up In addition, children in the per protocol analysis were censored on the day that they had their allocated treatment switched.

Diarrhoea and oedema resolution from baseline to day 90 was also assessed via Cox regression analysis. Results were identified as statistically significant when p<0.05 at 95% confidence interval (CI). To tackle missing data attributed to lost follow-up and absconded cases, we employed a Multiple Imputations analysis, assuming missingness at random as we felt there was sufficient variables in the dataset to explain the missingness. The non-monotone nature of the data, as revealed by Multiple Imputations pattern analysis, guided our approach. We utilized the Predictive Markov Chain Monte Carlo method with a Predictive Mean Matching model, generating 5 iterations for each missing data point.

Complete cases of MUAC, weight, WGV, WHZ, oedema, and diarrhoea status at baseline and day 1 served as predictors to generate imputations for MUAC, weight, Weight Gain Velocity (WGV), and WHZ variables on days 7, 28, and 90. The estimates for each missing value were derived by averaging the pooled results from the 5 iterations. The variables in the imputation model including the number of values imputed are described in Table S1 in the supplemental file.

### Sample-size estimation

The overall sample size was 160 children (80 per each study arm). A formal sample size was not calculated as the aim was to generate adequate data of a proof of principal that the modified nutritional feed provides clinical, physiological and biological evidence of benefit to the child in terms of nutritional rehabilitation. MUAC was selected as the primary criterion for nutritional recovery because it predicts mortality better and is less affected by oedema than other anthropometric measures^(25)^. Whilst a formal sample size was not calculated we were guided (for our primary endpoint) by a trial of antimicrobial prophylaxis, where in Kenyan children admitted with severe malnutrition the baseline mean MUAC was 10.6cm (SD 1.06) and by at 90 days 12.2cm (SD 1.35); a mean change of 1.6cm (SD 1.1) nutritional recovery at 90 days^(3)^. Since the study was designed to provide safety data and some indication of the likely efficacy of the modified feed compared to standard of care we thus aimed to study 160 children in total, which was realistic given the timeframe and funding available for the study.

## Results

**Figure 1:**
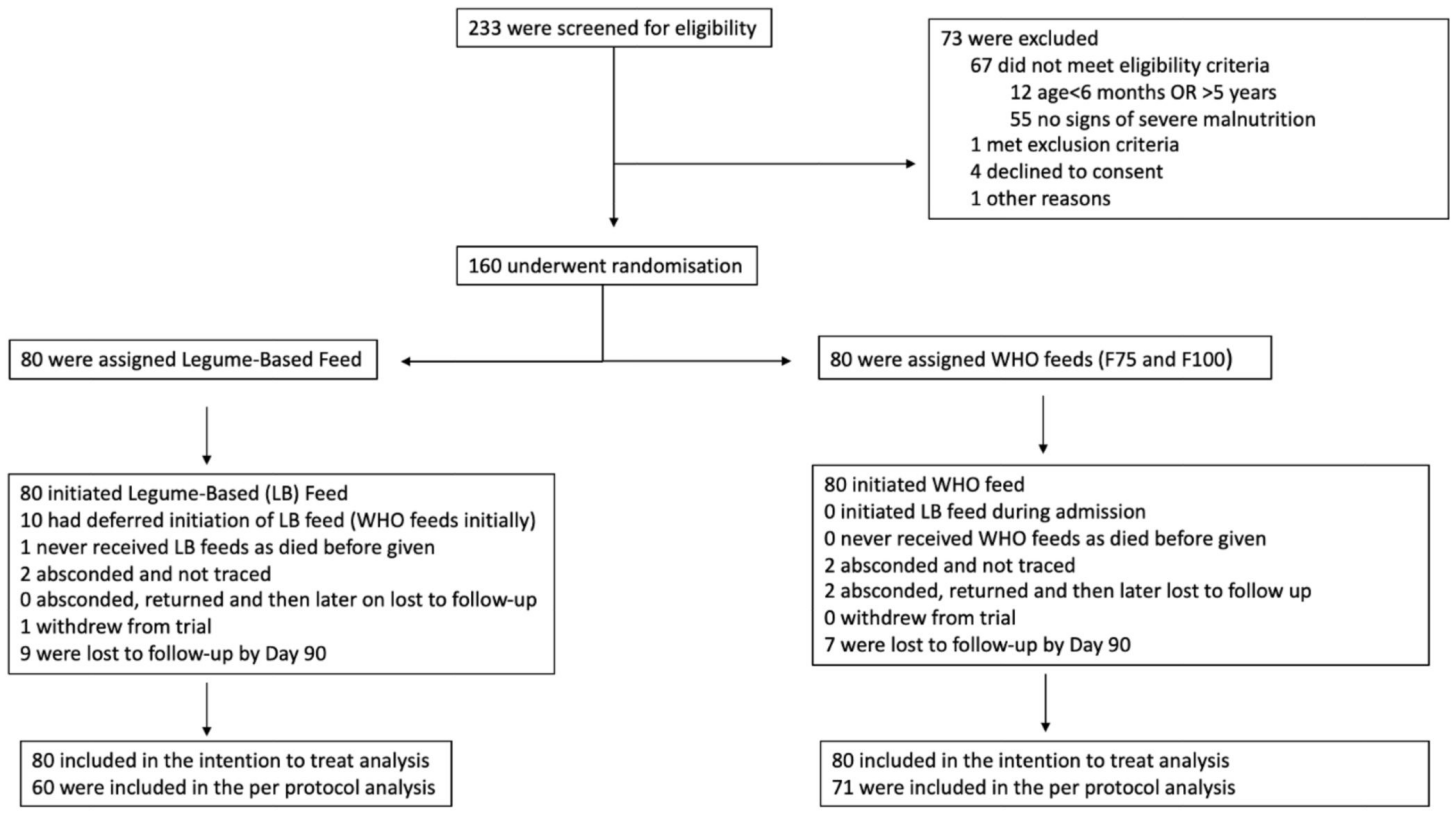
Trial Flow

Between 5^th^ July 2018 and 29^th^ May 2019 160 children, of a median age 17 (interquartile range, IQR 12-24) months were enrolled into the trial and randomised to legume-based feed (n=80) or WHO feeds (n=80). All children are included in the intention to treat analysis (Figure 1). Two (2.5%) and four (5%) of children in the legume and WHO arms respectively self-discharged (absconded) from hospital during the intial admission. Nine (11%) children in the legume and 7 (9%) in the WHO arm were lost to follow up (survival status at 90 days unknown). One child (LF arm) withdrew from the trial. (Figure 1 and Table S2). The last patient was followed up on 28^th^ August 2019^(26)^.

Baseline characteristics were well balanced between randomised groups, except oedematous malnutrition which was marginally more common in the legume feed (61% vs 53%) and of greater severity (generalised oedema) 10/49 (20%) vs 6/41 (15%) respectively (Table 1). Overall, diarrhoea was present in 42 (26%) children but respiratory distress (suspected pneumonia) 6 (4%) and HIV 7 (4%) were uncommon as was pre-exisiting developmental delay 9(6%). Biochemical markers of severity (severe hyponatraemia and hypokalaemia) were present in 30 (19%) and 18 (11%) children respectively. Many children had received antimicrobials prior to admission.

### Adherence to randomised feed and volume received

Detailed summaries of feed volumes and adherence up to day 14 hospitalisation are reported in Table S2. Overall, ten children randomised to legume feeds were switched to WHO feeds. From hospital admission (trial enrolment) through to day 14, 5 children switched from legume to WHO feeds. Two switched 2 within 48-hours of admission - as they unable tolerate non-liquid feeds since they developed severe decompensation (both died); Three switched between days 3-13 and a further 5 children switched feeds after day 14. Owing to the higher feed refusal in legume feed arm, the feed volumes given and percentage receiving the full amount were higher in the WHO arm on Day 0 and Day 1 however by Day 2 the total feed volume was similar between the two arms.

### Energy and protein intake

The daily summaries of energy and protein intake are reported in Table S3. Daily energy intake (reported in kilocalories) was slightly higher in the WHO arm on Day 0 and Day 1 but was similar after this. Overall, energy intake met the nutritional target in both groups at all time points. Protein content in the legume feeds were much higher between Days 0-3 but was equivalent beyond this timepoint. In both arms children met expected protein intake targets across all time points.

### Length of Hospital Admission

71/80 (88.8%) and 67/80 (83.4%) for the legume feed and WHO feed respectively recovered and were discharged home. Their median hospital length was 11 days (IQR 7) and 12 days (IQR 8) respectively. The length of admission for those surviving to discharge, deaths and absconders are summarised in box-and-whiskers plots and table (Supplemental Figure S1). The fatal cases on the legume and WHO feeds arms had a median hospital stay of 6.5 days (IQR 4.75) and 13 days (IQR 9.5) respectively.

### Outcomes

**Figure 2:**
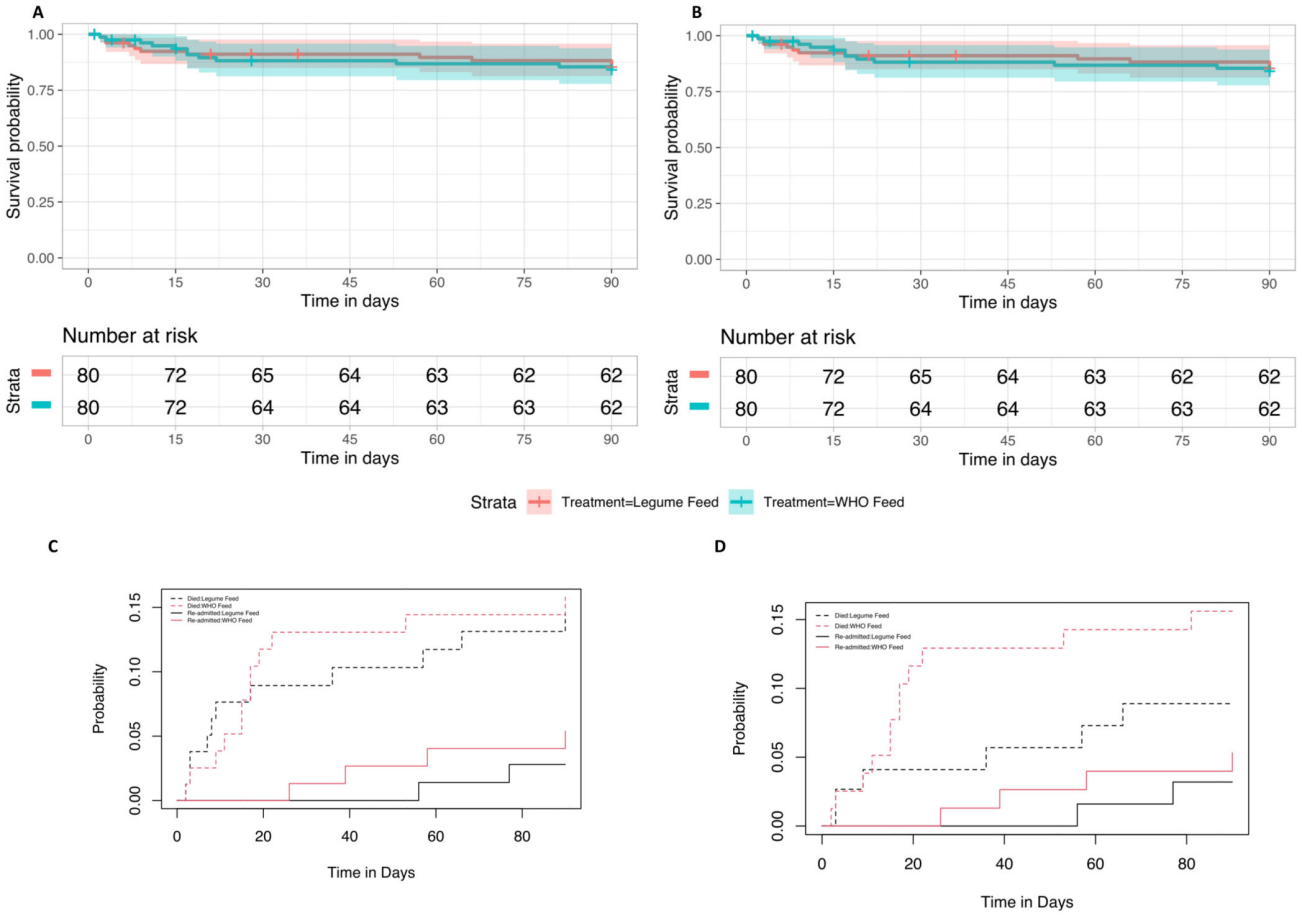
**a-d** Survival and Readmission Plots to Day 90 **2A:** Kapan Meier plot with 95% confidence intervals from an ITT analysis. **2B:** Kapan Meier plot with 95% confidence intervals from a PP analysis. **2C:** Competing risk analysis curves of re-admissions with mortality as a competing risk from ITT analysis 2**D:** Competing risk analysis curves of re-admissions with mortality as a competing risk from a PP analysis.

Primary and secondary endpoints are summarised in Table 2. By intention to treat there was no difference in change in MUAC by day 90 (primary endpoint) with a median change in centimeters of 1.1 (1.1 IQR) and 1.4 (1.40 IQR) for the legume feed arm and WHO feeds arm respectively (p=0.09). Day 90 mortality (co-primary endpoint) for legume feed and WHO feeds arms were similar: 11/80 (13.8%) and 12/80 (15%) respectively (also see Figure 2A (Kaplan Meier for mortality by intention to treat). Most deaths occurred within the initial period of hospitalisation 7/11 (64%) and 8/12 (67%) respectively, from complications of infection predominantly associated with diarrhoea and pneumonia comorbidities (Supplemental Table S4). Only 4 deaths occurred in children with HIV (3 in the WHO arm and 1 in the legume arm). As there were no differences in the baseline characteristics between arms in those treated per protocol (Supplemental Table S5) we conducted a per protocol analysis (which censored children who absconded during initial admission, those we were unable to ascertain survival status at Day 90 (lost to follow up) and children who switched from legume feed to WHO feeds). Per protocol there was little difference in the change in Day 90 MUAC from the intention to treat analysis. Day 90 mortality was lower in the legume feed arm 6/60 (10%) compared to 12/71 (17%) in the WHO arm but this was not statistically significant, hazard ratio 0.54 (95% confidence interval 0.20 – 1.45) p=0.22 (also see Figure 2B).

#### Secondary Endpoints

Few children achieved standard optimum of weight gain (>5g/kg) by day 90 in both arms, however time to resolution of oedema (Table 2) was similar in both arms. Diarrhoea resolved in most children before Day 7 with no difference between arms. Development of denovo diarrhoea was high (34/160 (21.3%) overall with no difference between arms. With respect to serious adverse events (safety endpoints) marginally more children in the WHO feeds arm were readmitted: 4 children (including one child twice) versus 2 in the legume feed arm (Table 2). The principal co-morbidities in the fatal cases mortality are summarised in Table S4. Readmission rates were assessed with competing risk analysis with mortality as a competing event. The analysis demonstrated that re-admissions were lower in the legume feed arm (3%) versus WHO feed arm (5%) in the ITT analysis (sub-hazard ratio 0.50 (95% CI 0.09 – 2.71), p=0.42. In the PP analysis redmissions were lower in the legume feed arm 2/69 (3%) versus WHO feed arm 4/71 (6%) sub-hazard ratio 0.58 (95% CI 0.11 – 3.14) p=0.53) (Figures 2C and 2D).

### Anthropometric changes in oedematous and non-oedematous children resolution

**Figure 3:**
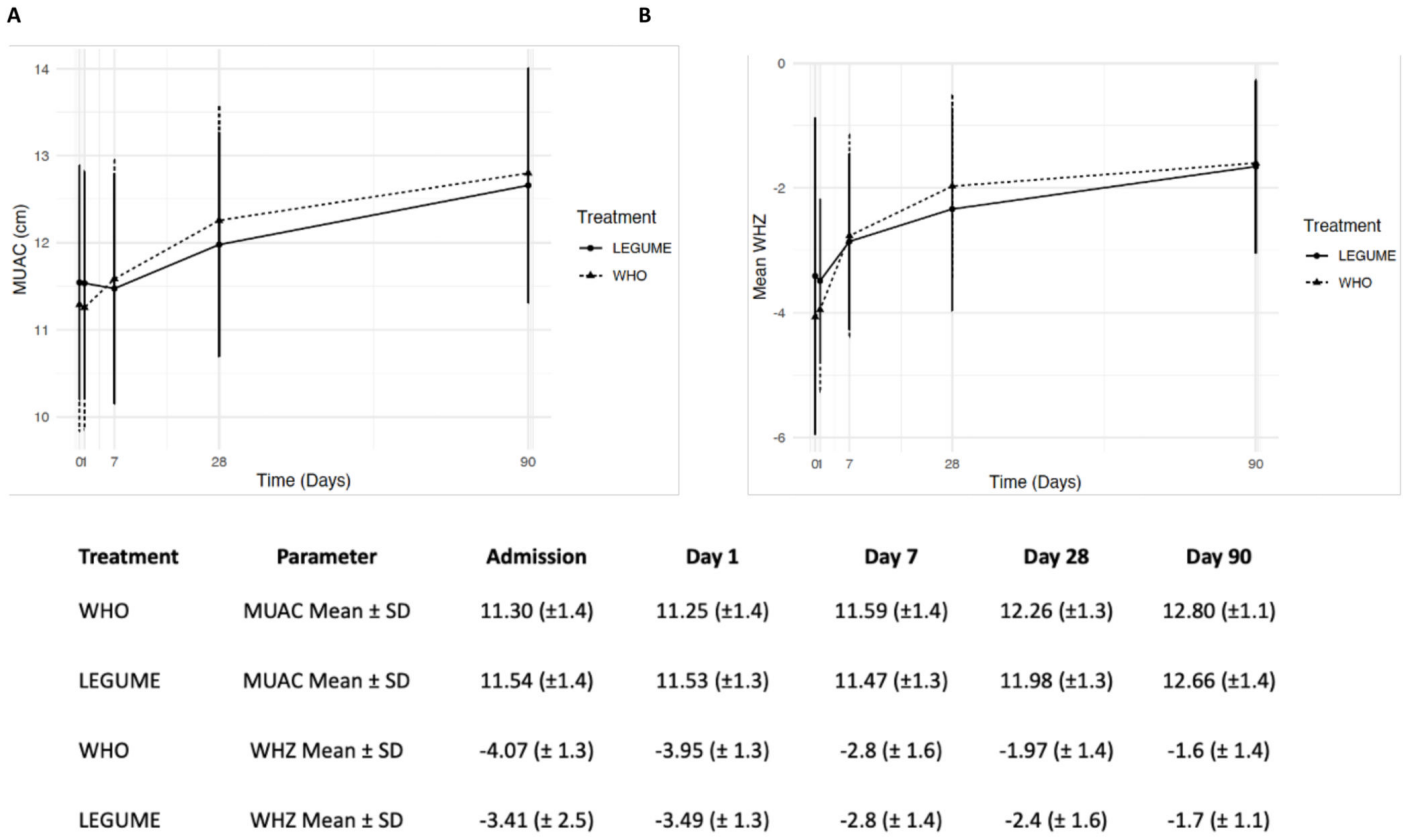
Mean (Standard Deviation) of mid-upper arm circumference (MUAC) and weight for height Z score (WHZ) from admission to Day 90

We were able to report detailed data on weight gain for study arms and for individual children overtime. Overall, mean (SD) MUAC and WHZ overtime are reported in Figure 3. In both study arms children transitioned from anthropometric parameters indicating severe malnutrition at trial entry to moderately and undernourished by Day 90. These parameters are summarised separately for oedematous and non-oedematous phenotypes (Supplemental Figure S2). The weight gain trajectory (gain, loss or maintenance) are reported over the follow up time period and stratified by presence of oedema at admission overall (Table 3) and for individuals (Supplementary Figure S2). We found little differences in early weight gains (to Day 7) in children without nutritional oedema. However, during this same period more children experienced weight loss in the legume arm in children presenting with oedema possibly due to the greater severity of oedema in the LF arm, which persisted to Day 28. By Day 90 weight gain occurred in 58/60 (97%) of children without oedema at baseline, whereas in children presenting with nutritional oedema 37/42 (88%) of the legume arm and 34/35 (97%) of the WHO arm had gained weight (p=0.613).

In an additional analysis we examined the number of children who would be classified as recovered (that is attaining a MUAC of >12.5cm) overtime and by study arm. In the ITT analysis at Day 28 26/71 (36.6%) in the WHO arm and 32/73 (44%) in legume feed arm had recovered. By Day 90 in the ITT analysis 43/68 (63.2%) in the WHO arm and 35/69 50.7%) in legume feed arm had recovered (Supplemental Table S6)

## Discussion

In this trial comparing two nutritional strategies in 160 Ugandan children admitted with severe acute malnutrition, including 57% with the kwashiorkor phenotype, we were able to demonstrate that legume-enriched feed provided similar anthropometric outcomes than children receiving the milk-based WHO formulae (F75 followed by F100). In general, the weight gain velocities were less than the recommended > 5g/kg/day for both strategies with oedematous children experiencing much lower growth velocities. Thus, by Day 90 most childrens’ anthropometric parameters were consistant with moderate to mild undernutrition. Mortality remained high, overall 14% (23 children) died within 20 days of admission. These were largely to due the complications of underlying infections (pneumonia and diarrhoea). In the intention to treat analyses we found no evidence for a difference in mortality between arms. In a per protocol analysis, we found Day 90 mortality was lower in the legume feed arm (10%) versus the standard feed arm (17.5%) and the rate of readmission by 90 days was less, 3% versus 5% respectively but neither of these finding were significant owing to the small sample size.

The trial was not directly powered to find a difference between the legume feed strategy and WHO feeds on patient-centred outcomes including de novo development of diarrhoea, mortality or readmissions. However, it demonstrated that this novel strategy provided similar anthropometric improvements to the WHO feed arm and with no evidence of harm. Owing to poor palatability of the nutritional paste (children preferred liquid-based feeds initially) a number of children switched early to WHO feeds which has implications for the future design of other legume based feeds. The baseline characteristics remain balanced between the two arms in the children included in the per protocol analysis population (when compared to children included in the ITT analysis, indicating no or minimal sampling bias (Supplemental Table S4).

Major limitations of the trial include the poor palatability of the legume feed especially during the initial few days resulted in children switching to WHO feeds. In addition, whilst we considered resolution of diarrhoea as an endpoint, we found the accuracy of reporting to be rather subjective. Parental reports of diarrhoea (defined as more than three loose stools) masked the spectrum of severity even though most diarrhoea largely resolved within a few days. Despite this, and in keeping with previous reports ^(27)^, the clinicians reported an additional 23% of children developed diarrhoea de novo during nutritional rehabilitation. Malnourished children with diarrhoea or developing diarrhoea in hospital are at risk of worse outcomes^(27)^. This has also been observed in children with uncomplicated severe malnutrition managed in the community^(27) (28)^. In this trial we found that the most common clinical complication contributing to in-patient deaths was diarrhoea in the WHO arm (5 patients) versus 1 patient in the legume feed arm. These findings and other emerging data indicates that there is substantial evidence of profound gut-barrier dysfunction, characterised by blunted villi^(29)^, inflammation and increased permeability^(30)^. Furthermore, children with severe malnutrition often having functional lactase, maltase and sucrase deficiency (the key F75/F100 disaccharides), which exacerbates diarrhoea, impairs vital nutritient uptake and nutritional recovery ^(10; 11)^. As a result current WHO formula have been adapted to reduce the sucrose load by incorporation of malodextran, which has a low risk of causing osmotic diarrhoea. Morever, attempts to modify the initial starter feed (F75) by reducing lactose and carbohydrate-load was examined in a Phase II trial which failed to improve outcomes (including time to stabilisation, diarrhoea and mortality) ^(5)^. Nor did providing elemental feeds (hypoallergenic and anti-inflammatory feeds) improve markers biomarkers of intestinal and systemic inflammation and mucosal integrity^(31)^. This indicates more radical revisions to the formula are required^(5)^. We had proposed that a lactose-free, fermentable carbohydrate-containing (chickpea) alternative^(22)^ may address the poor outcomes in this high risk group. Current research programmes in East Africa are expanding legume growth, including chickpeas, to improve the environmental impact of agriculture (nitrogen fixing)^(32)^, meaning that their use in nutritional feeds are both acceptable and readily available to local communities. Morover, chickpea-based follow-on formulae have been explored as a potential prevention for undernutrition ^(33)^.

Progress in the area of optimal nutritional feed for those who have been hospitalised with severe malnutrition has been very slow and piecemeal. Most field research conducted in Africa is largely in community-based programmes (uncomplicated severe malnutrition) often with good outcomes. Future research investigating whether innovative feeding strategies focusing on gut repair, optimizing the microbial environment as well as providing nutritional support after immediate recovery, could improve clinical outcomes compared to standard treatments (and less costly). This would be a substantive starting point to revise treatment guidelines. With respect to availability most nutritional feeds are largely manufactured remote from the continent or the communities mostly affected. Feed availability is dependent upon the international donors, at substantial costs, thus accessibility for local communities is low^(34)^. International non-governmental organisations have recognised that there is an unmet need and to develop them more locally as current formulations for inpatient and community feeds require dried milk, which is often limited, variable in quality and expensive. Research in this field in community programmes has also examine lower cost, milk-free plant based alternatives, showing non-inferiority to peanut-based RUTF^(35)^.

For children with severe and complicated malnutrition, this trial was the first step in providing some evidence that food products, which are all available locally in Uganda, could be used in future feeding strategies to address this unmet need directly and the research gap highlighted in the WHO report on RUTF feed composition^(36)^ Similar consultations for reviewing the content of inpatient feeds are lacking. What we have learned in this trial will enable us to design a legume feed which incorporates the essential minerals at the point of processing and that the initial feed should be liquid-based. Further trials in this area should focus on patient centred outcomes including mortality and readmissions as the their primary endpoints.

## Supplementary Material

Supplemental Tables and Figures

## Figures and Tables

**Table 1:** Baseline Characteristics

	Legume Based feed	WHO feeds (F75/F100)	Total
Participants, n	80	80	160
Age in months [Interquartile range)	18 [12,24.7)	17 [12,23.7]	17 [12,24]
Sex: Male (%)	44 (55)	39 (48.75)	83 (51.87)
**Nutritional status and history**			
Mid-upper arm circumference (MUAC), cm [IQR]	11.4 [10.5,12.2]	11.2 [10.5,12.2]	11.2 [10.5,12.2]
Weight for height Z score [IQR]	-3.79 [-4.4,-2.7]	-4.08 [-5,-2]	-4.0 [-4.6,-2.9]
Weight-for-height/length z score <-3	31 (39)	39 (49)	70 (44)
Oedema (kwashiorkor)	49 (61)	41 (51)	90 (56)
Oedema severity: severe/generalized	10/49 (20)	6/41 (15)	16/90 (18)
Signs of desquamation or flaky paint skin	20/80 (25)	15/80 (19)	35/160 (22)
Age when feeds introduced (months)	4 [3,6]	5 [3,6]	5 [3,6]
Currently breast feeding	21/80 (26.5)	24/80 (30)	45/160 (28)
Previous admission with severe malnutrion	5/80 (6)	4/80 (5)	9/160 (6)
**Complications at Presentation**			
History of fever	63/80 (79)	57/80 (71)	120/160 (75)
Fever (axillary temp) > 37.5°C	10/80 (12.5)	10/80 (12.5)	20/160 (12.5)
Cough	59/80 (74)	59/80 (74)	118/160 (46)
Indrawing or deep breathing	3/80 (4)	3/80 (4)	6/160 (4)
Vomiting	23/80 (29)	25/80 (31)	48/160 (19)
Diarrhoea	17/80 (21)	25/80 (31)	42/160 (26)
**Laboratory parameters**			
Hyponatraemia (<130 mmol/L)	17/78 (22)	13/80 (16)	30/158 (19)
Hypokalaemia (<3.0 mmol/L)	9/78 (11.5)	9/80 (11)	18/158 (11)
Hypoglycaemia (< 3mmol/dl)	4/80 (5)	1/80 (1)	5/160 (3)
Severe anaemia (Hb < 5g.dl)	2/78 (3)	1/79 (1)	3/157 (2)
Lactate > 2 mmols/L	39/68 (57)	36/71 (51)	75/139 (54)
Malaria film positive	15/80 (19)	7/80 (9)	22/160 (14)
HIV Antibody positive	1/80 (1)	6 /80 (7.5)	7/160 (4)
**Pre-extisting Conditions and ** **preadmission Treatments**			
Pulmonary Tuberculosis	1/80 (1)	2/80 (2.5)	3/160 (2)
Congenital Heart Disease	0	0	0
Cerebral Palsy/severe developmental delay	6/80 (7.5)	3/80 (4)	9/160 (6)
Currently taking antibiotics	25/80 (31)	28/80 (35)	33/160 (21)
Currently taking antimalarials	7/80 (9)	9/80 (11)	16/160 (10)
Currently taking antiretrovirals	1/80 (1)	6/80 (7.5)	7/160 (4)

Data are number (%) or median [interquartile range] unless otherwise specified.

**Table 2:** Primary and Secondary Outcomes including safety outcomes

	Legume	WHO		p*-value
**By ** ^α^ **Intention to Treat**	N=80	N=80	Hazard ratio (95% CI)	
**Co-primary outcomes**				
Median change (IQR) in MUAC Day 0 to Day 90	1.1 (1.1)cm	1.4 (1.40)cm		0.09*
Day 90 Mortality	11/80 (14%)	12/80 (15%)	0.91 (0.40-2.07)	0.83**
**Secondary Outcomes**				
Day 90 Weight gains of >5g/kg/day	5/69 (7%)	5/68 (5%)		0.45*
Day 90 Oedema resolution	42/49 (86%)	33/41 (81%)	1.27 (0.80-2.00)	0.29**
Day 90 Diarrhoea resolution	12/17 (71%)	20/25 (80%)	0.57 (0.28–1.16)	0.12**
Denovo development of diarrhoea (Day 2-14)	16/80 (20%)	18/80 (22.5%)	0.84 (0.43-1.66)	0.62**
Readmission to Day 90	2/80 (2.5%)	4/80 (6%)	0.50 (0.09-2.71)	0.42***
^β^ **Per-protocol**	N=60	N=71		
Median change (IQR) in MUAC Day 0 to Day 90	1.1 (1.30)cm	1.4 (1.35)cm		0.08*
Day 90 Mortality	6/60 (10%)	12/71 (17.5%)	0.54 (0.20-1.45)	0.22**
Day 90 Weight gain of >5g/kg/day	5/64 (8%)	5/68 (5%)		0.53*
Day 90 Oedema resolution	39/49 (79.5%)	33/41 (81%)	1.22 (0.82-2.07)	0.27**
Day 90 Diarrhoea resolution	12/15 (80%)	20/25 (80%)	0.57 (0.28- 1.17)	0.12**
Denovo development of diarrhoea (Day 2-14)	15/80 (18.8%)	18/80 (22.5%)	0.83 (0.42-1.64)	0.59**
Readmission to Day 90	2/60 (3%)	4/71 (6%)	0.58 (0.11-3.14)	0.53***

α **ITT Analysis:** Primary outcome results assessed based on their assigned randomised treatment (N=80), ignoring non-compliance with respect to the therapeutic feed intake.

β **PP Analysis:** Primary outcome results were assessed based on only the children who completed their originally allocated treatment.

* p-value estimated from a *Mann-Whitney U* test

** p-value estimated from unadjusted *Cox Regression* analysis

*** p-value represents Gray’s test from a *Competing risk analysis, with mortality as the competing risk*, and a sub-Hazard Ratio estimated.

**Table 3:** The number of children with weight -gain, -loss and -maintenance based stratified by oedematous status at baseline **(as per ITT)**

Time period	Non-Oedematous Children		Oedematous Children	
**Day 0- Day 7**	**WHO Feed**	**Legume Feed**	**WHO Feed**	**Legume Feed**
Weight Gain	29/38 (76%)	23/30 (77%)	24/40 (60%)	16/46 (35%)
Weight Loss	5/38 (13%)	4/30 (13%)	13/40 (32.5%)	25/46 (54%)
Maintenance	4/38 (11%)	3/30 (10%)	3/40 (7.5%)	5/46 (11%)
Statistic	0.137		0.022	
**Day 7- Day 28**				
Weight Gain	21/35 (60%)	20/29 (69%)	27/36 (75%)	25/44 (57%)
Weight Loss	11/35 (31%)	5/29 (17%)	7/36 (19%)	16/44 (36%)
Maintenance	3/35 (9%)	4/29(14%)	2/36 (6%)	3/44 (7%)
Statistic	0.075		0.027	
**Day 28- Day 90**				
Weight Gain	24/33 (73%)	23/27 (85%)	28/35 (80%)	34/42 (81%)
Weight Loss	7/33 (21%)	3/27 (11%)	5/35 (14%)	6/42 (14%)
Maintenance	2/33 (6%)	1/27 (4%)	2/35 (6%)	2/42 (5%)
Statistic	0.865		0.101	
**Day 0- Day 90**				
Weight Gain	32/33(97%)	26/27 (96%)	34/35 (97%)	37/42 (88%)
Weight Loss	0/33 (0%)	1/27 (4%)	1/35 (3%)	5/42 (12%)
Maintenance	1/33 (3%)	0/27 (0%)	0/35 (0%)	0/42 (0%)
Statistic	0.824		0.613	

* p-value: represents Mann-Whitney U statistical analysis

Weight gain: Weight _tB-tA_>0

Weight maintenance: Weight _tB-tA_= 0

Weight loss: Weight _tB-tA_ <0

## Data Availability

The datasets generated during the trial will be available upon reasonable request, following the publication of the trial results, from Prof. Kathryn Maitland (k.maitland@imperial.ac.uk). Anonymised data including clinical and anthropometric data will be made available. The data used in this research was collected subject to the informed consent of the participants. Access to the data will only be granted in line with that consent, subject to approval by the project ethics board and under a formal Data Sharing Agreement.

## Abbreviations

IQR: interquartile range
ITT: intention-to-treat
KWTRP: KEMRI-Wellcome Trust Research Programme
LF: Legume Feed
PP: per protocol
MIMBLE: Modifying Intestinal Microbiome with LEgume Based feed
MUAC: mid-upper arm circumference
SAE: serious adverse events
SCFA: short-chain fatty acids
WHO: World Health Organization
WGV: Weight Gain Velocity
WHZ: weight for height Z score
