# Legume-supplemented feed for children hospitalised with severe malnutrition: a phase II trial

**DOI:** 10.1017/S0007114524000837

**Published:** 2024-08-14

**Authors:** Kevin Walsh, Akglinta Kiosa, Peter Olupot-Olupot, Florence Alaroker, William Okiror, Margaret Nakuya, Tonny Tssenyondo, Denis Aromut, Bernard Charles Okalebo, Rita Muhindo, Ayub Mpoya, Elizabeth C. George, Gary S. Frost, Kathryn Maitland

**Affiliations:** 1 Division of Diabetes, Endocrinology and Metabolism, Imperial College, 6th Floor Commonwealth Building, Hammersmith Campus, DuCane Road, London W12, UK; 2 Department of Nutritional Sciences, School of Life Course & Population Sciences, Faculty of Life Sciences & Medicine, King’s College, London SE1 9NH, UK; 3 Mbale Clinical Research Institute, Busitema University Faculty of Health Sciences, Mbale Campus, Palissa Road, PO Box 1966, Mbale, Uganda; 4 Soroti Regional Referral Hospital, Hospital Road, PO Box 289, Soroti, Uganda; 5 Kenya Medical Research Institute (KEMRI)-Wellcome Trust Research Programme, Kilifi, Kenya; 6 Medical Research Council Clinical Trials Unit (MRC CTU) at University College London, London, UK; 7 Imperial College, Department of Infectious Disease and Institute of Global Health and Innovation, Faculty of Medicine, Imperial College, London, UK

**Keywords:** Severe malnutrition, Clinical trial, Legume-based feeds, African children

## Abstract

Children hospitalised with severe malnutrition have high mortality and readmission rates post-discharge. Current milk-based formulations target restoring ponderal growth but not the modification of gut barrier integrity or microbiome which increases the risk of gram-negative sepsis and poor outcomes. We propose that legume-based feeds rich in fermentable carbohydrates will promote better gut health and improve overall outcomes. We conducted an open-label phase II trial at Mbale and Soroti Regional Referral Hospitals, Uganda, involving 160 children aged 6 months to 5 years with severe malnutrition (mid-upper arm circumference (MUAC) < 11·5 cm and/or nutritional oedema). Children were randomised to a lactose-free, chickpea-enriched legume paste feed (LF) (*n* 80) *v*. WHO standard F75/F100 feeds (*n* 80). Co-primary outcomes were change in MUAC and mortality to day 90. Secondary outcomes included weight gain (> 5 g/kg/d), *de novo* development of diarrhoea, time to diarrhoea and oedema resolution. Day 90 MUAC increase was marginally lower in LF *v*. WHO arm (1·1 cm (interquartile range (IQR) 1·1) *v*. 1·4 cm (IQR 1·40), *P* = 0·09); day 90 mortality was similar (11/80 (13·8 %) *v*. 12/80 (15 %), respectively, OR 0·91 (95 % CI 0·40, 2·07), *P* = 0·83). There were no differences in any of the other secondary outcomes. Owing to initial poor palatability of the LF, ten children switched to WHO feeds. Per-protocol analysis indicated a trend to lower day 90 mortality and readmission rates in the LF (6/60 (10 %) and 2/60(3 %)) *v*. WHO feeds (12/71(17·5 %) and 4/71(6 %)). Further refinement of LF and clinical trials are warranted, given the poor outcomes in children with severe malnutrition.

Severe malnutrition remains a frequent cause of hospitalisation in African children. It is associated with high in-hospital mortality rates of about 20 %^(1,2)^ and poor long-term outcomes^(3,4)^. Clinical trials addressing infection prophylaxis^(3)^ or modification of the standard recommended WHO feed^(5)^ have failed to improve the poor outcomes. Milk-based feeds recommended for the management of severe malnutrition (called F75 and F100) result in nutritional (anthropometric) recovery in survivors (the current gold standard of success). Nevertheless, this poorly predicts short- and long-term outcomes^(6)^, including increased risk of life-threatening events (death and/or re-hospitalisation with pneumonia or diarrhoea) in the 12 months following initial admission^(4,7)^. A phase II trial examining other formulations compared a feed with reduced lactose and carbohydrate load in the starter feed compared with standard formula (F75). This did not demonstrate improvement in outcomes, indicating that more radical approaches are required in the design of nutritional feeds^(5)^.

There are multiple lines of evidence indicating that several domains of gut function are aberrant in children with severe malnutrition. Intestinal atrophy^(8,9)^ results in functional loss of brush border disaccharidases (lactase, maltase and sucrose)^(10,11)^ which exacerbates diarrhoea and impairs recovery. Moreover, there is a significant relative microbiota immaturity and high levels of pathogenic flora in children with severe malnutrition which are only partially ameliorated following 3 weeks of standard nutritional interventions^(12)^. We hypothesised that intestinal mucosal integrity and gut microbial diversity can be restored in severe malnutrition by providing substrates that induce fermentation in the gastrointestinal tract^(13)^. Fermentable carbohydrates can improve the balance of normal gut microbes and positively influence the immunological and metabolic function of the gut^(14,15)^. Carbohydrates that escape digestion in the gastrointestinal tract (resistant starch and dietary fibre) induce favourable changes in colonic microbiota fermentation^(16)^. These lead to the generation of SCFA which have a positive influence on gut integrity and nutritional health by improving energy yield, modulation of colonic pH, production of vitamins and the stimulation of gut homoeostasis, including anti-pathogen activities^(17,18)^. We tested this hypothesis in a pilot trial (Modifying Intestinal Microbiome by Legume-Based fEeds: MIMBLE 1 PACTR201805003381361) which compared cowpea-supplemented standard nutritional formulae to standard WHO formulae (F75/F100)^(19)^. We demonstrated the feed was safe and palatable and resulted in equivalent weight and mid-upper arm circumference (MUAC) gain compared with standard WHO formulae (F75/F100)^(19)^. In the standard WHO feed arm, faecal microbiota diversity showed very little change over the 28-d intervention and no change in major phyla. Furthermore, the SCFA concentrations on admission were approximately a third of the concentration of those reported in healthy African infants^(20)^. However, in the standard WHO feed arm, but not the cowpea arm, there was a suppression of the SCFA propionate and butyrate at day 7 (to about 1/10th of the normal concentrations), a period when the children are at high risk of mortality. We suspect that the suppression of SCFA at day 7 may have been due to the use of antibiotics, which recovered once antibiotic treatments were stopped. *In vitro* batch culture (in an artificial colon) of the WHO milk feed (F75/100) demonstrated no impact on the gut microbiome or microbiological diversity, whereas the cowpea-enhanced feeds lead to increases in bifidobacteria (that has been linked to improved epithelial integrity^(21)^) and diversity. Since there were no differences in diarrhoea (frequency and resolution) or other clinical endpoints between the feeds, we made further modifications to the feed and developed, with a UK food manufacturer, a lactose-free, fermentable carbohydrate-containing alternative feed (MIMBLE 2 feed)^(22)^ swapping cowpea for chickpea. Here, we report the phase II clinical trial which compared a chickpea-supplemented lactose-free feed to standard (WHO) milk feeds on a range of endpoints. The trial was registered with ISRCTN 10309022 on 23/05/2018.

## Methods

Between 5 July 2018 and 28 August 2019, we conducted an open-label, proof-of-principle randomised controlled trial on the paediatric wards in two sites (Mbale and Soroti Regional Referral Hospitals) in Eastern Uganda. The trial was designed to evaluate the safety and efficacy of lactose-free chickpea-based nutritional formulae compared with standard milk-based feeds recommended by WHO (control).

### Screening, randomisation and blinding

Children with suspected severe malnutrition were clinically assessed for eligibility and exclusion criteria. Children aged 6 months to 5 years hospitalised with severe malnutrition were eligible for trial enrolment. Severe malnutrition was defined as either marasmus (defined by MUAC < 11·5 cm) ‘and/or’ kwashiorkor (defined as symmetrical pitting oedema involving at least the feet irrespective of weight-for-height *Z* score (WHZ) or MUAC). In children with life-threatening complications, where prior written consent from parents/legal guardians could not be obtained, ethics committees approved parental verbal assent and deferred written informed consent as soon as practicable^(23)^. Otherwise, informed written consent was obtained from parents or guardians before randomisation. Children with severe malnutrition with a co-morbidity at very high risk of death, for example, malignant disease or terminal illness, or a parent/guardian not willing to consent were not eligible for this trial.

An independent data manager, based at KEMRI-Wellcome Trust Research Programme (KWTRP), Kenya, generated the sequential randomisation list, using permuted blocks. This sequence was used, by a study administrator at KWTRP, Kilifi, Kenya, to prepare randomisation cards with the treatment allocation which were sequentially numbered and sealed in opaque envelopes, each signed across the seal ensuring allocation concealment. In the hospitals in Uganda, randomisation was done by the study clinician using the numbered envelopes sequentially which contained the randomised feed strategy. Nurses/doctors were unblinded to study intervention; laboratory tests were assayed blinded. Children were randomly assigned 1:1 to either legume-based paste feed (investigational) or F75/F100 feeds (control) recommended by WHO.

### Study interventions

The details of the content, development and nutrient profiles of the legume-based feed have been previously reported^(22)^ Briefly, the ingredients were lactose-free skimmed milk powder (7·25 %), rapeseed oil (11·5 %), gram flour (10 %), sugar (9 %) and water. All ingredients were sourced from established EU suppliers and passed all required safety tests for human consumption as appropriate for each ingredient (contaminants, pesticides, toxins, bacterial contamination, etc.). The final feed contained (per 100 ml) 200 kcal, 18 g total carbohydrate, 5·6 g protein, 12 g fat and 0·4 g resistant starch. The final product matched the macronutrient profile of double-concentrated F100 adhered to all relevant legislation regulating infant foods. Chickpeas were selected as the source of resistant starch, since they are widely grown and eaten throughout Africa. Micronutrient (vitamins and minerals) content could not be matched in this ready-to-use product, so this was replaced at the point of feeding. The detailed feeding protocol for legume-based is detailed in the published protocol^(24)^ Children in the control arm received F75 and F100 as per WHO recommendations.

### Study procedures

Children were managed in general paediatric ward. A structured clinical record documented relevant clinical, examination and laboratory baseline assessment. Nutritional feeds were given per the published protocol^(24)^. Briefly, for those in the control arm (WHO feeds), initially 130 ml/kg/d F75 therapeutic milk was given at 4-h intervals over the day until the child was stabilised and demonstrating appetite. At this point, they transitioned to 4-h F100 therapeutic milk at the same rate and increased by 10 ml per feed, until a maximum rate of 200 ml/kg/d was achieved. Legume feeds (LF) were provided as a paste for 4 h at 45–50 g/kg/d (or 35–40 g/kg/d if oedematous). With additional water per feed provided starting at 105–110 ml/kg/d. Feed weight and additional water volume were adjusted daily in accordance with increasing weight (using a feed volume/weight calculation chart). Once clinically stable, the feed weight increased by 5 g/feed until a maximum of 100 g/kg/d. Mineral mix was added to the water for children in the legume arm (as WHO F100 and F75 formulae already contained mineral mix). Thus, the quantity of LF provided matched the total amounts of energy and protein that would be received in the control arm, and additional water was provided to match the fluid received. If the child took less than 80 % of feed volume/weight for two consecutive feeds, despite attempts with a spoon or syringe, then children were offered nasogastric tube feeding. Children in the legume strategy who could not initially tolerate non-fluid diet were switched to the WHO standard F75 feed and could then return to the legume strategy when non-liquid feeds were tolerated. All feed volumes and problems with feeding were recorded on standard proforma. Other standard treatments were prescribed, including antimalarials and antibiotics, following national guidelines.

Children were reviewed twice daily to discharge (generally about day 14). On consenting to the MIMBLE 2 study, patients/parents/guardians agreed to remain in the hospital for a minimum of 7 d but preferably 14 d (based on both the WHO and Ugandan Ministry of Health guidelines). Patients were permitted to leave earlier if they had no oedema if applicable, good weight gain and MUAC > 12·5 cm. Treatment with control/experimental treatment will be for 14 d duration, followed by standard treatment outpatient as required. This includes the provision of ready-to-use therapeutic feeds until the child has recovered, defined by a WHZ > –2. At discharge, children were back to the community nutrition programmes where they were reviewed as per standard country guidelines. Children were reviewed by the study teams for clinical status and anthropometric status at 28- and 90-d post-randomisation. Serious adverse events were actively solicited which included prolongation of hospital admission, readmission, mortality and suspected allergic reaction to the feed.

### Endpoints

The co-primary endpoint was change in MUAC at day 90 and mortality at day 90. Secondary outcome measures included the change of weight and achieving a weight gain of > 5 g/kg/d by day 28 and day 90, parent-reported *de novo* development of diarrhoea (> 3 loose stools/d) and time to resolution of diarrhoea, time to oedema resolution and presence of oedema at days 28 and 90, and the number of serious adverse events (prolongation of hospital admission, readmission, mortality and suspected allergic reaction to the feed to day 90).

### Statistical analysis

Clinical data were analysed by using IBM Statistical Package for Social Sciences (SPSS) for Windows version 28 (IBM Corp.) and R and R Studio Core Team (2022). Calculation of WHZ was performed by using the online WHO Anthro Survey Analyser. Primary and secondary outcomes were analysed on an intention-to-treat (ITT) and per-protocol (PP) basis. PP analysis was defined as the analysis that included children who received and successfully completed their allocated treatment upon randomisation, during their hospital stay, and their survival status was known until study discharge (day 90).

Differences in feed provision, anthropometric and clinical characteristics between the two groups on different follow-up days were evaluated using the non-parametric Mann–Whitney *U* test due to the non-normal distribution of anthropometric data. For categorical data, such as diarrhoea and oedema status at baseline and on days 7, 28 and 90, *χ* ^2^ analysis was employed. Cox regression analysis and Kaplan–Meier survival curves were employed to assess mortality and recovery differences between treatments, with competing risks regression analysis for readmissions, employing the Fine and Gray competing risk regression model, with the competing risk of death. Children were censored if they absconded (left the hospital or nutritional follow-up against medical advice) or lost to follow-up In addition, children in the PP analysis were censored on the day that they had their allocated treatment switched.

Diarrhoea and oedema resolution from baseline to day 90 was also assessed via Cox regression analysis. Results were identified as statistically significant when *P* < 0·05 at 95 % CI. To tackle missing data attributed to lost follow-up and absconded cases, we employed a multiple-imputation analysis, assuming missingness at random as we felt there were sufficient variables in the dataset to explain the missingness. The non-monotone nature of the data, as revealed by multiple-imputation pattern analysis, guided our approach. We utilised the predictive Markov chain Monte Carlo method with a predictive mean matching model, generating five iterations for each missing data point.

Complete cases of MUAC, weight, weight gain velocity, WHZ, oedema and diarrhoea status at baseline and day 1 served as predictors to generate imputations for MUAC, weight, weight gain velocity and WHZ variables on days 7, 28 and 90. The estimates for each missing value were derived by averaging the pooled results from the five iterations. The variables in the imputation model including the number of values imputed are described in online Supplementary Table S1.

### Sample-size estimation

The overall sample size was 160 children (80 per study arm). A formal sample size was not calculated as the aim was to generate adequate data of a proof of principal that the modified nutritional feed provides clinical, physiological and biological evidence of benefit to the child in terms of nutritional rehabilitation. MUAC was selected as the primary criterion for nutritional recovery because it predicts mortality better and is less affected by oedema than other anthropometric measures^(25)^. Whilst a formal sample size was not calculated, we were guided (for our primary endpoint) by a trial of antimicrobial prophylaxis, where in Kenyan children admitted with severe malnutrition the baseline mean MUAC was 10·6 cm (sd 1·06) and by at 90 days 12·2 cm (sd 1·35), a mean change of 1·6 cm (sd 1·1) nutritional recovery at 90 d^(3)^. Since the study was designed to provide safety data and some indications of the likely efficacy of the modified feed compared with the standard of care, we thus aimed to study 160 children in total, which was realistic given the time frame and funding available for the study.

## Results

**Fig. 1.**
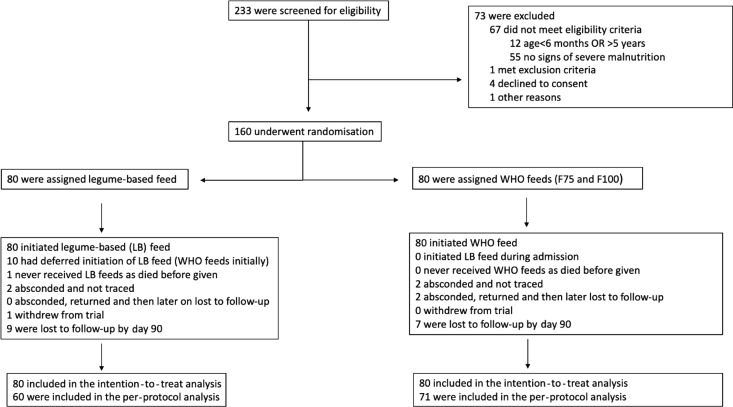
Trial flow.

Between 5 July 2018 and 29 May 2019, 160 children of a median age of 17 (interquartile range, (IQR) 12–24) months were enrolled in the trial and randomised to legume-based feed (*n* 80) or WHO feeds (*n* 80). All children are included in the ITT analysis (Fig. 1). Two (2·5 %) and four (5 %) of children in the legume and WHO arms, respectively, self-discharged (absconded) from the hospital during the initial admission. Nine (11 %) children in the legume and seven (9 %) in the WHO arm were lost to follow-up (survival status at 90 d unknown). One child (LF arm) withdrew from the trial (Fig. 1 and online Supplementary Table S2). The last patient was followed up on 28 August 2019^(26)^.

Baseline characteristics were well balanced between randomised groups, except oedematous malnutrition which was marginally more common in the LF (61 % *v*. 53 %) and of greater severity (generalised oedema) 10/49 (20 %) *v*. 6/41 (15 %), respectively (Table 1). Overall, diarrhoea was present in forty-two (26 %) children, but respiratory distress (suspected pneumonia) in six (4 %) and HIV in seven (4 %) were uncommon as was pre-exisiting developmental delay 9(6 %). Biochemical markers of severity (severe hyponatraemia and hypokalaemia) were present in thirty (19 %) and eighteen (11 %), children respectively. Many children had received antimicrobials prior to admission.

**Table 1.** Baseline characteristics (Numbers and percentages; median values and interquartile ranges)

	Legume-based feed		WHO feeds (F75/F100)		Total	
	*n*	%	*n*	%	*n*	%
Participants, *n*	80		80		160	
Age in months						
Median	18		17		17	
Interquartile range	12, 24·7		12, 23·7		12, 24	
Sex: male (%)	44	55	39	48·75	83	51·87
Nutritional status and history						
Mid-upper arm circumference (MUAC) (cm)						
Median	11·4		11·2		11·2	
Interquartile range	10·5, 12·2		10·5, 12·2		10·5, 12·2	
Weight-for-height *Z* score						
Median	–3·79		–4·08		–4·0	
Interquartile range	–4·4, –2·7		–5, –2		–4·6, –2·9	
Weight-for-height/length *Z* score < –3	31	39	39	49	70	44
Oedema (kwashiorkor)	49	61	41	51	90	56
Oedema severity: severe/generalised	10/49	20	6/41	15	16/90	18
Signs of desquamation or flaky paint skin	20/80	25	15/80	19	35/160	22
Age when feeds introduced (months)						
Median	4		5		5	
Interquartile range	3, 6		3, 6		3, 6	
Currently breast-feeding	21/80	26·5	24/80	30	45/160	28
Previous admission with severe malnutrition	5/80	6	4/80	5	9/160	6
Complications at presentation						
History of fever	63/80	79	57/80	71	120/160	75
Fever (axillary temp) > 37·5°C	10/80	12·5	10/80	12·5	20/160	12·5
Cough	59/80	74	59/80	74	118/160	46
Indrawing or deep breathing	3/80	4	3/80	4	6/160	4
Vomiting	23/80	29	25/80	31	48/160	19
Diarrhoea	17/80	21	25/80	31	42/160	26
Laboratory parameters						
Hyponatraemia (< 130 mmol/l)	17/78	22	13/80	16	30/158	19
Hypokalaemia (< 3·0 mmol/l)	9/78	11·5	9/80	11	18/158	11
Hypoglycaemia (< 3 mmol/dl)	4/80	5	1/80	1	5/160	3
Severe anaemia (Hb < 5 g.dl)	2/78	3	1/79	1	3/157	2
Lactate > 2 mmols/l	39/68	57	36/71	51	75/139	54
Malaria film positive	15/80	19	7/80	9	22/160	14
HIV antibody positive	1/80	1	6/80	7·5	7/160	4
Pre-existing conditions and pre-admission treatments						
Pulmonary tuberculosis	1/80	1	2/80	2·5	3/160	2
Congenital heart disease	0		0		0	
Cerebral palsy/severe developmental delay	6/80	7·5	3/80	4	9/160	6
Currently taking antibiotics	25/80	31	28/80	35	33/160	21
Currently taking antimalarials	7/80	9	9/80	11	16/160	10
Currently taking antiretrovirals	1/80	1	6/80	7·5	7/160	4

Data are number (%) or median (interquartile range) unless otherwise specified.

### Adherence to randomised feed and volume received

Detailed summaries of feed volumes and adherence up to day 14 hospitalisation are reported in online Supplementary Table S2. Overall, ten children randomised to LF were switched to WHO feeds. From hospital admission (trial enrolment) to day 14, five children switched from legume to WHO feeds. Two switched within 48 h of admission – as they are unable to tolerate non-liquid feeds since they developed severe decompensation (both died); three switched between days 3 and 13, and further five children switched feeds after day 14. Owing to the higher feed refusal in the LF arm, the feed volumes given and the percentage receiving the full amount were higher in the WHO arm on day 0 and day 1; however, by day 2, the total feed volume was similar between the two arms.

### Energy and protein intake

The daily summaries of energy and protein intake are reported in online Supplementary Table S3. Daily energy intake (reported in kilocalories) was slightly higher in the WHO arm on day 0 and day 1 but was similar after this. Overall, energy intake met the nutritional target in both groups at all time points. Protein content in the LF was much higher between days 0 and 3 but was equivalent beyond this time point. In both arms, children met expected protein intake targets across all time points.

### Length of hospital admission

71/80 (88·8 %) and 67/80 (83·4 %) for the LF and WHO feed, respectively, recovered and were discharged home. Their median hospital length was 11 d (IQR 7) and 12 d (IQR 8), respectively. The length of admission for those surviving to discharge, deaths and absconders are summarised in box-and-whisker plots and table (online Supplementary Fig. S1). The fatal cases on the legume and WHO feeds arms had a median hospital stay of 6·5 d (IQR 4·75) and 13 d (IQR 9·5), respectively.

### Outcomes

**Fig. 2.**
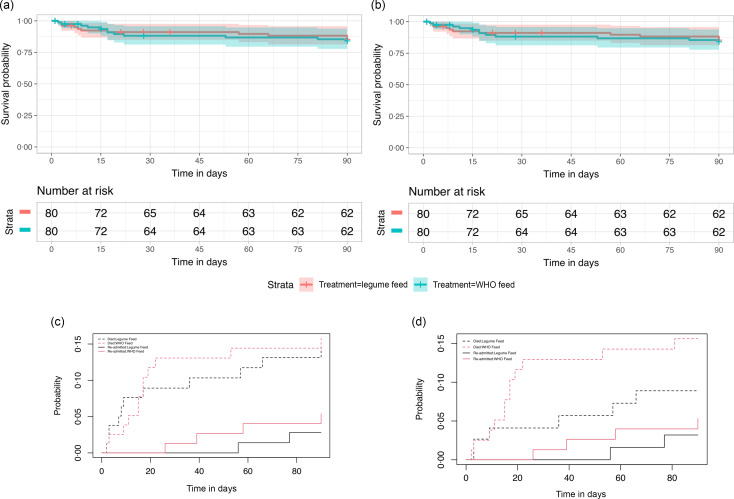
(a)–(d) Survival and readmission plots to day 90. (a) Kaplan–Meier plot with 95 % CI from an ITT analysis. (b) Kaplan–Meier plot with 95 % CI from a PP analysis. (c) Competing risk analysis curves of readmissions with mortality as a competing risk from ITT analysis. (d) Competing risk analysis curves of readmissions with mortality as a competing risk from a PP analysis. ITT intention-to-treat; PP per protocol.

Primary and secondary endpoints are summarised in Table 2. By ITT analysis, there was no difference in change in MUAC by day 90 (primary endpoint) with a median change in centimetres of 1·1 (1·1 IQR) and 1·4 (1·40 IQR) for the LF arm and WHO feeds arm, respectively (*P* = 0·09). Day 90 mortality (co-primary endpoint) for LF and WHO feeds arms were similar: 11/80 (13·8 %) and 12/80 (15 %), respectively (also see Fig. 2(a) (Kaplan–Meier curve for mortality by ITT analysis). Most deaths occurred within the initial period of hospitalisation 7/11 (64 %) and 8/12 (67 %), respectively, from complications of infection predominantly associated with diarrhoea and pneumonia co-morbidities (online Supplementary Table S4). Only four deaths occurred in children with HIV (three in the WHO arm and one in the legume arm). As there were no differences in the baseline characteristics between arms in those treated per protocol (online Supplementary Table S5), we conducted a PP analysis (which censored children who absconded during initial admission, children those we were unable to ascertain survival status at day 90 (lost to follow-up) and children who switched from LF to WHO feeds). Per protocol, there was little difference in the change in day 90 MUAC from the ITT analysis. Day 90 mortality was lower in the LF arm 6/60 (10 %) compared with 12/71 (17 %) in the WHO arm, but this was not statistically significant (hazard ratio 0·54 (95 % CI 0·20 – 1·45), *P* = 0·22) (also see Fig. 2(b)).

**Table 2.** Primary and secondary outcomes including safety outcomes (Numbers and percentages; median values and interquartile ranges; hazard ratios and 95 % CI)

	Legume		WHO		Hazard ratio	95 % CI	*P* *
By intention-to-treat†	*n* 80		*n* 80				
Co-primary outcomes							
Median change in MUAC day 0 to day 90	1·1 cm		1·4 cm				0·09*
IQR	1·1 cm		1·40 cm				
Day 90 mortality	11/80	14 %	12/80	15 %	0·91	0·40, 2·07	0·83**
Secondary outcomes							
Day 90 weight gains of > 5 g/kg/d	5/69	7 %	5/68	5 %			0·45*
Day 90 oedema resolution	42/49	86 %	33/41	81 %	1·27	0·80, 2·00	0·29**
Day 90 diarrhoea resolution	12/17	71 %	20/25	80 %	0·57	0·28, 1·16	0·12**
*De novo* development of diarrhoea (day 2–14)	16/80	20 %	18/80	22·5 %	0·84	0·43, 1·66	0·62**
Readmission to day 90	2/80	2·5 %	4/80	6 %	0·50	0·09, 2·71	0·42***
Per-protocol‡	*n* 60		*n* 71				
Median change (IQR) in MUAC day 0 to day 90	1·1 cm		1·4 cm				0·08*
IQR	1·30 cm		1·35 cm				
Day 90 mortality	6/60	10 %	12/71	17·5 %	0·54	0·20, 1·45	0·22**
Day 90 weight gain of > 5 g/kg/d	5/64	8 %	5/68	5 %			0·53*
Day 90 oedema resolution	39/49	79·5 %	33/41	81 %	1·22	0·82, 2·07	0·27**
Day 90 diarrhoea resolution	12/15	80 %	20/25	80 %	0·57	0·28, 1·17	0·12**
*De novo* development of diarrhoea (day 2–14)	15/80	18·8 %	18/80	22·5 %	0·83	0·42, 1·64	0·59**
Readmission to day 90	2/60	3 %	4/71	6 %	0·58	0·11, 3·14	0·53***

MUAC, mid-upper arm circumference; IQR, interquartile range; ITT intention-to-treat; PP per protocol.

* *P*-value estimated from a Mann–Whitney *U* test.

** *P*-value estimated from unadjusted Cox regression analysis.

*** *P*-value represents Gray’s test from a competing risk analysis, with mortality as the competing risk, and a sub-hazard ratio estimated.

† ITT analysis: primary outcome results assessed based on their assigned randomised treatment (*n* 80), ignoring non-compliance with respect to the therapeutic feed intake.

‡ PP analysis: primary outcome results were assessed based on only the children who completed their originally allocated treatment.

#### Secondary endpoints

Few children achieved the standard optimum of weight gain (> 5 g/kg) by day 90 in both arms; however, time to resolution of oedema (Table 2) was similar in both arms. Diarrhoea resolved in most children before day 7 with no difference between arms. Development of *de novo* diarrhoea was high (34/160 (21·3 %)) overall with no difference between arms. With respect to serious adverse events (safety endpoints), marginally more children in the WHO feeds arm were readmitted: four children (including one child twice) *v*. two in the LF arm (Table 2). The principal co-morbidities in the fatal cases mortality are summarised in online Supplementary Table S4. Readmission rates were assessed with competing risk analysis with mortality as a competing event. The analysis demonstrated that readmissions were lower in the LF arm (3 %) *v*. WHO feed arm (5 %) in the ITT analysis (sub-hazard ratio 0·50 (95 % CI 0·09, 2·71), *P* = 0·42). In the PP analysis, readmissions were lower in the LF arm 2/69 (3 %) *v*. WHO feed arm 4/71 (6 %) (sub-hazard ratio 0·58 (95 % CI 0·11, 3·14), *P* = 0·53) (Fig. 2(c) and (d)).

### Anthropometric changes in oedematous and non-oedematous children resolution

**Fig. 3.**
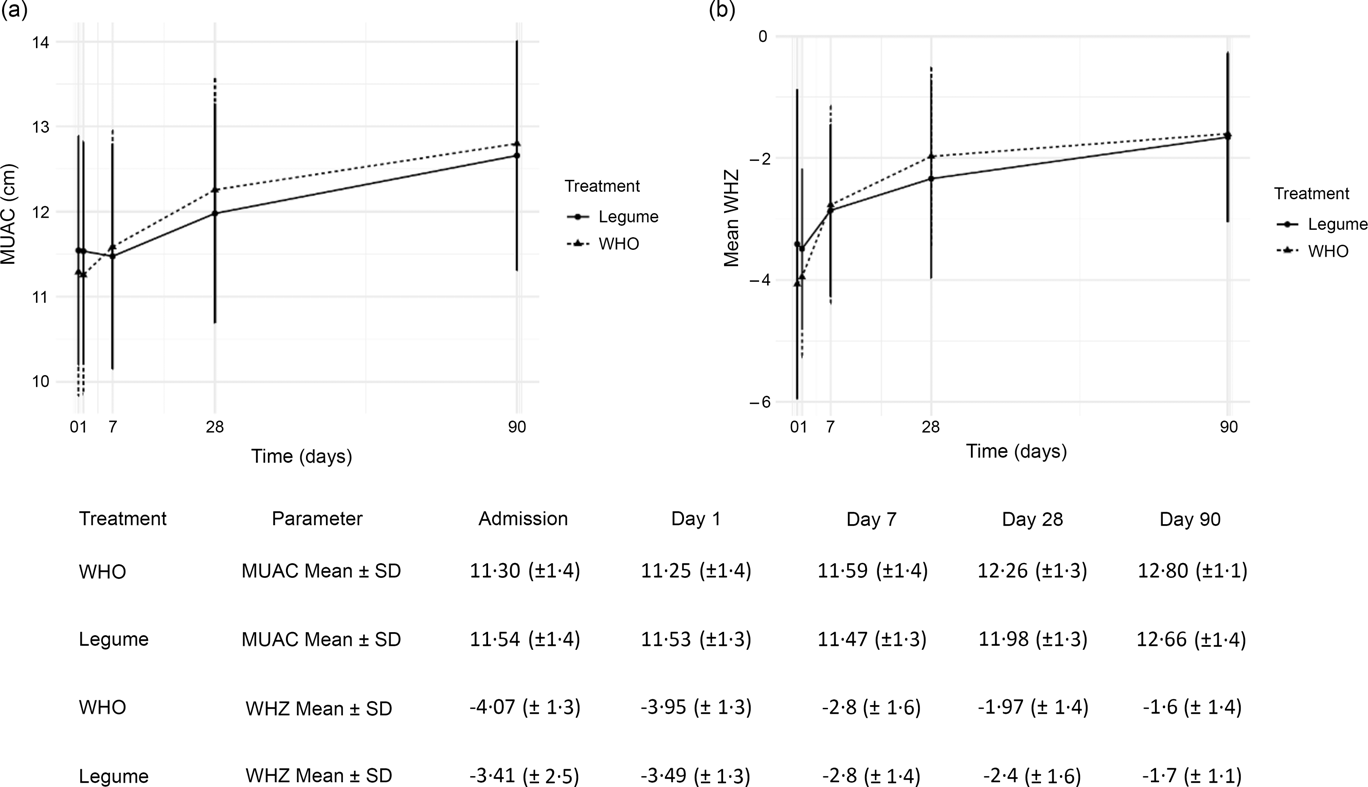
Mean and standard deviation of mid-upper arm circumference (MUAC) and weight-for-height *Z* score (WHZ) from admission to day 90.

We were able to report detailed data on weight gain for study arms and for individual children over time. Overall, mean and standard deviations of MUAC and WHZ over time are reported in Fig. 3. In both study arms, children transitioned from anthropometric parameters indicating severe malnutrition at trial entry to moderately and undernourished by day 90. These parameters are summarised separately for oedematous and non-oedematous phenotypes (online Supplementary Fig. S2). The weight gain trajectory (gain, loss or maintenance) is reported over the follow-up time period and stratified by the presence of oedema at admission overall (Table 3) and for individuals (online Supplementary Fig. S2). We found little differences in early weight gains (to day 7) in children without nutritional oedema. However, during this same period, more children experienced weight loss in the legume arm in children presenting with oedema possibly due to the greater severity of oedema in the LF arm, which persisted to day 28. By day 90, weight gain occurred in 58/60 (97 %) of children without oedema at baseline, whereas in children presenting with nutritional oedema 37/42 (88 %) of the legume arm and 34/35 (97 %) of the WHO arm had gained weight (*P* = 0·613).

**Table 3.** The number of children with weight gain, weight loss and weight maintenance-based stratified by oedematous status at baseline (as per ITT) (Numbers and percentages)

	Non-oedematous children				Oedematous children			
Time period	WHO feed		Legume feed		WHO feed		Legume feed	
Day 0–day 7	*n*	%	*n*	%	*n*	%	*n*	%
Weight gain	29/38	76 %	23/30	77 %	24/40	60 %	16/46	35 %
Weight loss	5/38	13 %	4/30	13 %	13/40	32·5 %	25/46	54 %
Maintenance	4/38	11 %	3/30	10 %	3/40	7·5 %	5/46	11 %
Statistic*	0·137				0·022			
Day 7–day 28								
Weight gain	21/35	60 %	20/29	69 %	27/36	75 %	25/44	57 %
Weight loss	11/35	31 %	5/29	17 %	7/36	19 %	16/44	36 %
Maintenance	3/35	9 %	4/29	14 %	2/36	6 %	3/44	7 %
Statistics*	0·075				0·027			
Day 28–day 90								
Weight gain	24/33	73 %	23/27	85 %	28/35	80 %	34/42	81 %
Weight loss	7/33	21 %	3/27	11 %	5/35	14 %	6/42	14 %
Maintenance	2/33	6 %	1/27	4 %	2/35	6 %	2/42	5 %
Statistics*	0·865				0·101			
Day 0–day 90								
Weight gain	32/33	97 %	26/27	96 %	34/35	97 %	37/42	88 %
Weight loss	0/33	0 %	1/27	4 %	1/35	3 %	5/42	12 %
Weight maintenance	1/33	3 %	0/27	0 %	0/35	0 %	0/42	0 %
Statistics*	0·824				0·613			

Weight gain: Weight _tB-tA_ > 0.

Weight maintenance: Weight _tB-tA_ = 0.

Weight loss: Weight _tB-tA_ < 0.

* *P*-value represents Mann–Whitney *U* statistical analysis.

In an additional analysis, we examined the number of children who would be classified as recovered (i.e. attaining a MUAC of > 12·5 cm) over time and by the study arm. In the ITT analysis at day 28, 26/71 (36·6 %) in the WHO arm and 32/73 (44 %) in the LF arm had recovered. By day 90 in the ITT analysis, 43/68 (63·2 %) in the WHO arm and 35/69 50·7 %) in the LF arm had recovered (online Supplementary Table S6)

## Discussion

In this trial comparing two nutritional strategies in 160 Ugandan children admitted with severe acute malnutrition, including 57 % with the kwashiorkor phenotype, we were able to demonstrate that legume-enriched feed provided similar anthropometric outcomes as children receiving the milk-based WHO formulae (F75 followed by F100). In general, the weight gain velocities were less than the recommended > 5 g/kg/d for both strategies with oedematous children experiencing much lower growth velocities. Thus, by day 90, most children’s anthropometric parameters were consistent with moderate to mild undernutrition. Mortality remained high, overall 14 % (23 children) died within 20 d of admission. These were largely due to the complications of underlying infections (pneumonia and diarrhoea). In the ITT analysis, we found no evidence for a difference in mortality between arms. In a PP analysis, we found day 90 mortality was lower in the LF arm (10 %) *v*. the standard feed arm (17·5 %) and the rate of readmission by 90 d was less, 3 % *v*. 5 %, respectively, but neither of these findings were significant owing to the small sample size.

The trial was not directly powered to find a difference between the LF strategy and WHO feeds on patient-centred outcomes, including *de novo* development of diarrhoea, mortality or readmissions. However, it demonstrated that this novel strategy provided similar anthropometric improvements to the WHO feed arm with no evidence of harm. Owing to poor palatability of the nutritional paste (children preferred liquid-based feeds initially), a number of children switched early to WHO feeds which has implications for the future design of other legume-based feeds. The baseline characteristics remain balanced between the two arms in the children included in the PP analysis population (when compared with children included in the ITT analysis), indicating no or minimal sampling bias (online Supplementary Table S4).

Major limitations of the trial include the poor palatability of the LF, especially during the initial few days resulted in children switching to WHO feeds. In addition, whilst we considered the resolution of diarrhoea as an endpoint, we found the accuracy of reporting to be rather subjective. Parental reports of diarrhoea (defined as more than three loose stools) masked the spectrum of severity, even though most diarrhoea largely resolved within a few days. Despite this, and in keeping with previous reports^(27)^, the clinicians reported an additional 23 % of children developed diarrhoea *de novo* during nutritional rehabilitation. Malnourished children with diarrhoea or who developed diarrhoea in hospital are at risk of worse outcomes^(27)^. This has also been observed in children with uncomplicated severe malnutrition managed in the community^(27,28)^. In this trial, we found that the most common clinical complication contributing to inpatient deaths was diarrhoea in the WHO arm (five patients) *v*. one patient in the LF arm. These findings and other emerging data indicate that there is substantial evidence of profound gut barrier dysfunction, characterised by blunted villi^(29)^, inflammation and increased permeability^(30)^. Furthermore, children with severe malnutrition often have functional lactase, maltase and sucrose deficiency (the key F75/F100 disaccharides), which exacerbates diarrhoea and impairs vital nutrient uptake and nutritional recovery^(10,11)^. As a result, the current WHO formulae have been adapted to reduce the sucrose load by incorporating maltodextrin, which has a low risk of causing osmotic diarrhoea. Attempts to modify the initial starter feed (F75) by reducing lactose and carbohydrate load, examined in a phase II trial, failed to improve outcomes (including time to stabilisation, diarrhoea and mortality)^(5)^. Nor did providing elemental feeds (hypoallergenic and anti-inflammatory feeds) improve biomarkers of intestinal and systemic inflammation and mucosal integrity^(31)^. This indicates that more radical revisions to the formula are required^(5)^. We proposed that a lactose-free, fermentable carbohydrate-containing (chickpea) alternative^(22)^ may address the poor outcomes in this high-risk group. Current programmes in East Africa are expanding legume growth, including chickpeas, to improve the environmental impact of agriculture (nitrogen-fixing)^(32)^, meaning that their uses in nutritional feeds are both acceptable and readily available to local communities. Chickpea-based follow-on formulae have been explored as a potential prevention for undernutrition^(33)^.

Progress in the area of optimal nutritional feed for those who have been hospitalised with severe malnutrition has been very slow and piecemeal. Most research conducted in Africa is largely in community-based programmes (uncomplicated severe malnutrition) often with good outcomes. Future research investigating whether innovative feeding strategies focusing on gut repair, optimising the microbial environment as well as providing nutritional support after immediate recovery could improve clinical outcomes compared with standard treatments (and less costly). This would be a substantive starting point to revise treatment guidelines. With respect to availability, most nutritional feeds are largely manufactured remote from the continent or the communities mostly affected. Feed availability is dependent on the international donors, at substantial costs; thus, accessibility for local communities is low^(34)^. International non-governmental organisations have recognised that there is an unmet need to develop them more locally as current formulations for inpatient and community feeds require dried milk, which is often limited, variable in quality and expensive. Research in this field in community programmes has also examined lower-cost, milk-free plant-based alternatives, showing non-inferiority to peanut-based Ready-to-use therapeutic food (RUTF)^(35)^.

For children with severe and complicated malnutrition, this trial was the first step in providing some evidence that food products, which are all available locally in Uganda, could be used in future feeding strategies to address this unmet need directly and the research gap highlighted in the WHO report on RUTF composition^(36)^ Similar consultations for reviewing the content of inpatient feeds are lacking. What we have learned in this trial will enable us to design an LF that incorporates the essential minerals at the point of processing and that the initial feed should be liquid-based. Further trials in this area should focus on patient-centred outcomes, including mortality and readmissions as their primary endpoints.

## Supporting information

Walsh et al. supplementary material Walsh et al. supplementary material

## Supplementary material

For supplementary material accompanying this paper visit https://doi.org/10.1017/S0007114524000837
