# Supplementary material for: Legume-supplemented feed for children hospitalised with severe malnutrition: A Phase II trial
Source: Br J Nutr. Author manuscript; Available in PMC 2024 Sep 20. (PMC7616506; doi:10.1017/S0007114524000837)
Supplement: Supplementary file 1 [file EMS195350-supplement-Supplemental_Tables_and_Figures.pdf]

Supplemental Tables and Figures

Table S1 Multiple imputations (MI) analysis Table
Table S2 Daily feed Provision for children receiving standard feed (WHO) and legume based feeds (LF)
Table S3 Daily energy (in kilocalories) and protein (in grams) and proportion meeting target intakes
Table S4 Mortality in children summarising underlying complications/conditions
Table S5 Baseline characteristics comparisons for WHO and legume feeds for intention to treat (ITT) and per protocol (PP) analysis.
Table S6 Proportion of children achieving MUAC > 12.5cm at each time point and by study arm

Figure S1 Status of children and length of hospital stay by study arm
Figure S2 Individual weight line plots based on oedematous status from baseline

Table S1 Multiple Imputation listing

Number of Multiple Imputation Analysis values on MUAC, Weight & Height, Oedema and

Total N of Imputed Missing Data by Treatment by Time-point					
Treatment	**D0**	**D1**	**D7**	**D28**	**D90**
**WHO**	0	1	3	5	9
**Legume**	0	0	2	7	11

***Treatment, Weight, Height and MUAC diarrhoea and oedema at baseline were used in the model as complete variables.**

Table S2 Daily feed Provision for children receiving standard feed (WHO) and legume-based feeds (LF)

	**n/N (%) that Received any feed**		**Total N of Deceased Participants**		**γTotal N switched to WHO feed**	**n/N (%) Received fully prescribed feed**		**Chi-square p-value**
Days	WHO	LF	WHO	LF	Legume Group only	WHO	LF	
0	80/80 (100)	*78/80 (97.5)	0	0	0	61/80 (76.25)	36/78 (46.1)	<0.001
1	**79/80 (98.75)	^78/80 (97.5)	0	0	1	72/79 (91.1)	56/78 (71.8)	0.003
2	79/80 (98.75)	α72/79 (91.1)	0	1	4	69/79 (87.3)	53/72 (73.6)	0.053
3	78/79 (98.7)	70/77 (90.9)	1	3	5	65/78 (83.3	53/70 (75.7)	0.344
4	***75/78 (96.1)	69/77 (89.6)	2	3	6	66/75 (88.0)	59/69 (85.5)	0.845
5	~73/78 (93.6)	68/77 (88.3)	2	3	7	65/73 (89)	56/68 (82.35)	0.370
6	73/78 (93.6)	68/76 (89.5)	2	4	7	64/73 (87.7)	51/68 (75)	0.085
7	70/74 (94.6)	β66/75 (88)	2	5	6	60/70 (85.7)	49/66 (74.2)	0.144
8	62/66 (93.9)	55/65 (84.6)	2	5	7	47/62 (75.8)	43/55 (78.2)	0.933
9	#50/55 (90.9)	52/62 (83.8)	2	5	6	41/50 (77.4)	40/52 (76.9)	0.697
10	43/48 (89.5)	45/55 (81.8)	3	5	6	34/43 (79.1)	35/46 (76.1)	1
11	35/40 (87.5)	40/49 (81.6)	3	5	5	27/35 (90)	25/40 (62.5)	0.262
12	30/35 (85.7)	32/42 (76.1)	4	5	4	25/30 (83.3)	25/32 (78.1)	0.844
13	27/32 (84.3)	31/42 (73.8)	4	5	5	22/27 (81.5)	23/31 (74.2)	0.728
14	24/29 (82.7)	26/39 (66.6)	4	5	5	21/24 (87.5)	19/26 (73.1)	0.358
Notes	*****2 children refused feed on admission and one out of the two later on was started on F75 on day 1 and died the next day<br>****** Child absconded from hospital<br>***2 Children absconded from hospital.<br>**~** 1 child absconded.<br># 1 child absconded.<br>**^** 1 child absconded.<br>**α** 1 child absconded.<br>**β** 1 child absconded.<br>γ The number varies with deaths occurring on children after they switched feeds.							

Table S3. Daily energy (in kilocalories) and protein (in grams) and proportion meeting target intakes

	**Median (IQR) of Energy Intake in Kcal**		**Median (IQR) Intake of energy vs target %**		**Mann Whitney U (p-value)**	**Median (IQR) of Protein Intake in g**		**Median of protein intake compared to target %**		**Mann Whitney U (p-value)**
Days	WHO	LF	WHO	LF	Between Treatments	WHO	LF	WHO	LF	Between Treatments
0	553.1 (208.13)	*499.8 (235.2)	100 (0)	95.6 (24.4)	<0.001	6.6 (2.5)	13.8 (6.5)	100 (0)	95.6 (24.4)	<0.001
1	**563 (225)	^592.3 (214.8)	100 (0)	100 (2)	0.002	6.8 (2.7)	16.5 (6)	100 (0)	100 (2)	0.002
2	585 (247)	α609.6 (203.6)	100 (0)	100 (1.2)	0.007	6.8 (2.7)	16.8 (8.3)	100 (0)	100 (1.2)	0.007
3	660 (267.75)	643.1 (363.7)	100 (0)	100 (0.5)	0.171	13.1 (16.2)	17.7 (10)	100 (0)	100 (0.5)	0.171
4	***730 (287)	670.6 (415.5)	100 (0)	100 (0)	0.411	20.9 (18.2)	18.5 (11.4)	100 (0)	100 (0)	0.411
5	~780 (376.7)	713.2 (303.3)	100 (0)	100 (0)	0.138	22.6 (19.5)	19.6 (8.4)	100 (0)	100 (0)	0.138
6	810 (337.5)	731.5 (397.7)	100 (0)	100 (0.9)	0.063	23.5 (10.5)	20.2 (10.9)	100 (0)	100 (0.9)	0.064
7	840 (333.7)	β783.3 (458.2)	100 (0)	100 (1.1)	0.228	24.4 (12.7)	21.6 (12.6)	100 (0)	100 (1.1)	0.228
8	810 (380.6)	853 (477.5)	100 (0.4)	100 (0)	0.385	23.1 (19.1)	23.5 (13.2)	100 (0.4)	100 (0)	0.385
9	#836.5 (397)	853.4 (548.6)	100 (0)	100 (0)	0.538	23.9 (20.3)	22.5 (14.9)	100 (0)	100 (0)	0.538
10	810 (397)	975.4 (601)	100 (0)	100 (0)	0.876	23.5 (20.2)	26.9 (16.6)	100 (0)	100 (0)	0.876
11	840 (339)	817.8 (548.1)	100 (0)	100 (8)	0.196	24.4 (19.4)	22.5 (15.1)	100 (0)	100 (8)	0.196
12	885 (391.7)	877.8 (603.5)	100 (0)	100 (0)	0.560	25.2 (17.2)	24.2 (16.6)	100 (0)	100 (0)	0.560
13	870 (397)	853.4 (694.9)	100 (0)	100 (1)	0.468	23.5 (21)	23.5 (19.1)	100 (0)	100 (1)	0.468
14	851 (399.2)	829 (521.2)	100 (0)	100 (4)	0.007	19.4 (20.2)	22.8 (14.4)	100 (0)	100 (4)	0.007
Notes	*2 children refused feed on admission and one out of the two later on was started on F75 on day 5 up to day 12 of discharge.<br>** Child absconded from hospital.<br>***2 Children absconded from hospital.<br>~ 1 child absconded.<br># 1 child absconded.<br>^ 1 child absconded.<br>α 1 child absconded.<br>β 1 child absconded.									

Table S4 Mortality in children summarising underlying complications/conditions

**Treatment**	WHO	LF	Comment
**Mortality All**	12	11	
Inpatient deaths	8	7	
Major clinical syndrome associated with inpatient death			
Diarrhoea	5	1	
LRTI	1	4	
Sepsis other	1 (Measles)	1 (malaria)	
Tuberculosis	1		
Other		1	Pancytopenia
Post discharge deaths	4	4	
(HIV-related)	(3)	(1)	
**Readmissions**			
Readmission and death	2	1	
Readmission only	2	1	

Post discharge deaths WHO arm:
Readmission/death: 1 diarrhoea 1 sepsis; 2 deaths in community unknown
Post discharge deaths Legume feed:
Readmission/death 1 diarrhoea; Deaths in community: 1 diarrhoea, relapse of kwashiorkor, measles

Table S5 Baseline characteristics comparisons for WHO and legume feeds for intention to treat (ITT) and per protocol (PP) analysis.

**Characteristic**	**By intenstionto treat**		**Per-protocol Analysis**	
	**Legume feed**	**WHO feeds (F75/F100)**	**Legume feed**	**WHO feeds (F75/F100)**
Participants, n	80	80	60	71
Median age in months [Interquartile range)	18 [12.7)	17 [11.7]	17 [12.2]	17 [11.5]
Sex: Male (%)	44 (55)	39 (48.75)	33 (51.6)	34 (53.12)
**Nutritional status and history**				
Median mid-upper arm circumference, cm [IQR]	11.4 [1.7]	11.2 [1.7]	11.4 [1.32]	11.2 [1.8]
Weight-for-height/length z score <-3	31 (39)	39 (49)	23 (38)	39 (49)
Weight for height Z score [IQR]	-3.79 [1.7]	-4.08 [3]	-4.05 [1.57]	-4.19 [2.08]
Oedema (kwashiorkor)	49 (61)	41 (51)	37 (62)	39 (44)
Severe/generalized Oedema	10/49 (20)	6/41 (15)	6/37 (16)	6/39 (15)
Desquamation or flaky paint skin	20/80 (25)	15/80 (19)	14/60 (23)	16/71 (22.5)
Age when feeds introduced (months)	4 [3]	5 [3]	4 [3]	5 [2.5]
Currently breast feeding	21/80 (26.5)	24/80 (30)	15/60 (25)	21/71 (30)
Previous admission with SAM	5/80 (6)	4/80 (5)	4/60 (7)	4/71 (6)
**Complications at Presentation**				
History of fever	63/80 (79)	57/80 (71)	48/60 (80)	50/71 (70)
Fever (axillary temp) > 37.5°C	10/80 (12.5)	10/80 (12.5)	9/60 (15)	8/71 (11)
Cough	59/80 (74)	59/80 (74)	45/60 (75)	54/71 (76)
Indrawing or deep breathing	3/80 (4)	3/80 (4)	2/60 (3)	3/71 (4)
Vomiting	23/80 (29)	25/80 (31)	13/60 (22)	30/71 (30)
Diarrhoea	17/80 (21)	25/80 (31)	11/60 (18)	19/71 (27)
**Laboratory parameters**				
Hyponatraemia (<130 mmol/L)	17/78 (22)	13/80 (16)	16/60 (27)	11/71 (15.5)
Hypokalaemia (<3.0 mmol/L)	9/78 (11.5)	9/80 (11)	6/60 (10)	8/71 (11)
Hypoglycaemia (< 3mmol/dl)	5/80 (6)	1/80 (1)	3/60 (5)	0
Severe anaemia (Hb < 5g.dl)	2/78 (3)	1/79 (1)	1/60 (2)	1/71 (1)
Lactate > 2 mmols/L	39/68 (57)	36/71 (51)	22/60 (37)	28/71 (39)
Malaria film positive	15/80 (19)	7/80 (9)	12/60 (20)	6/71 (8.5)
HIV Antibody positive	1/80 (1)	6 /80 (7.5)	1/60 (2)	5 /71 (7)
**Pre-existing Conditions /preadmission treatments**				
Pulmonary Tuberculosis	1/80 (1)	2/80 (2.5)	1/60 (2)	2/71 (3)
Congenital Heart Disease	0	0	0	0
Cerebral Palsy/severe developmental delay	6/80 (7.5)	3/80 (4)	4/60 (7)	3/71 (4)
Currently taking antibiotics	25/80 (31)	28/80 (35)	19/60 (32)	26/71 (37)
Currently taking antimalarials	7/80 (9)	9/80 (11)	7/60 (12)	7/71 (10)
Currently taking antiretrovirals	1/80 (1)	6/80 (7.5)	1/60 (2)	5/71 (7)

Data are number (%) or median [interquartile range] unless otherwise specified.
$^{\alpha}$ **ITT Analysis:** Primary outcome results assessed based on their assigned randomised treatment (N=80), ignoring non-compliance with respect to the therapeutic feed intake. $^{\beta}$ **PP Analysis:** Primary outcome results were assessed based on only the children.

Table S6 Proportion of children achieving MUAC > 12.5cm at each time point and by study arm

By intention to treat	Baseline	D7	D28	D90
WHO	13/80 (16.25%)	12/78 (15.4%)	26/71 (36.6%)	43/68 (63.2%)
LF	14/80 (17.5%)	11/76 (14.5%)	32/73 (44%)	35/69 50.7%)

Per Protocol	Baseline	D7	D28	D90
WHO	13/80 (16.25%)	12/78 (15.4%)	26/71 (36.6%)	43/68 (63.2%)
LF	1/80 (17.5%)	12/70 (17.0%)	21/67 (31.1%)	32/64 50%)

Figure S1 Status of children and length of hospital stay by study arm.

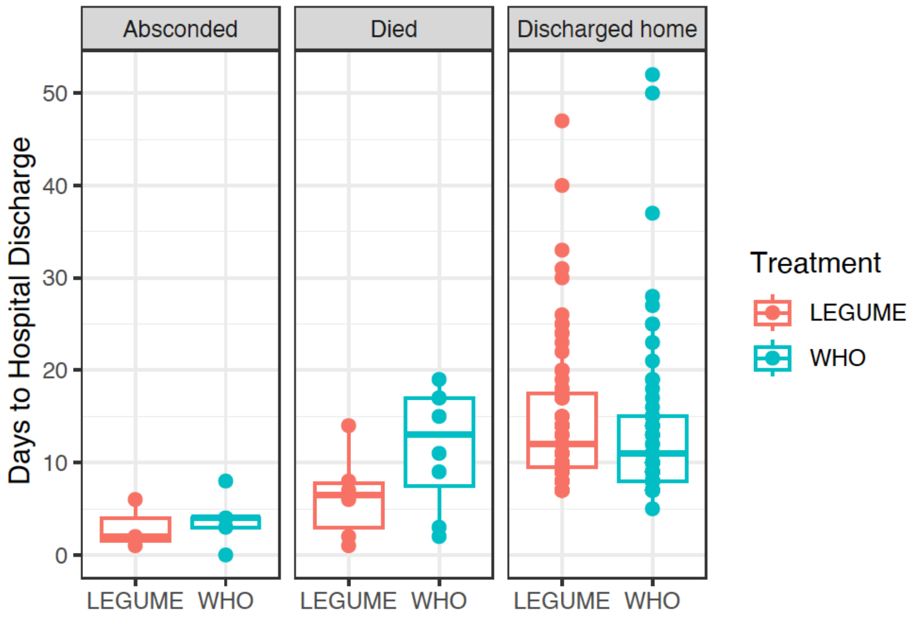

Treatment	Status	N	Median	IQR
LEGUME	Absconded	3	2	2.5
LEGUME	Died	6	6.5	4.75
LEGUME	Discharged home	71	12	8
WHO	Absconded	5	4	1
WHO	Died	8	13	9.5
WHO	Discharged home	67	11	7

Figure S2 Individual weight line plots based on oedematous status from baseline.

a) Non-oedematous at baseline

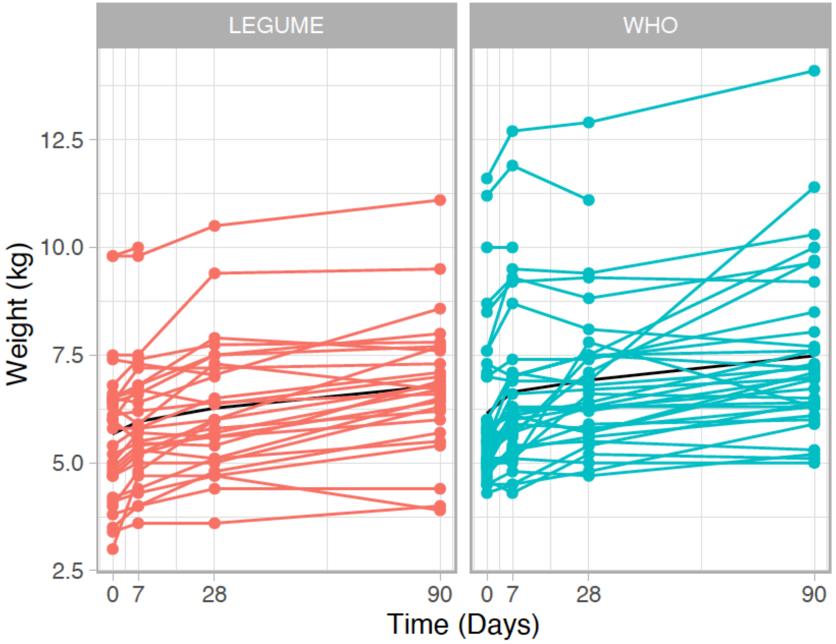

b) Oedema present at baseline

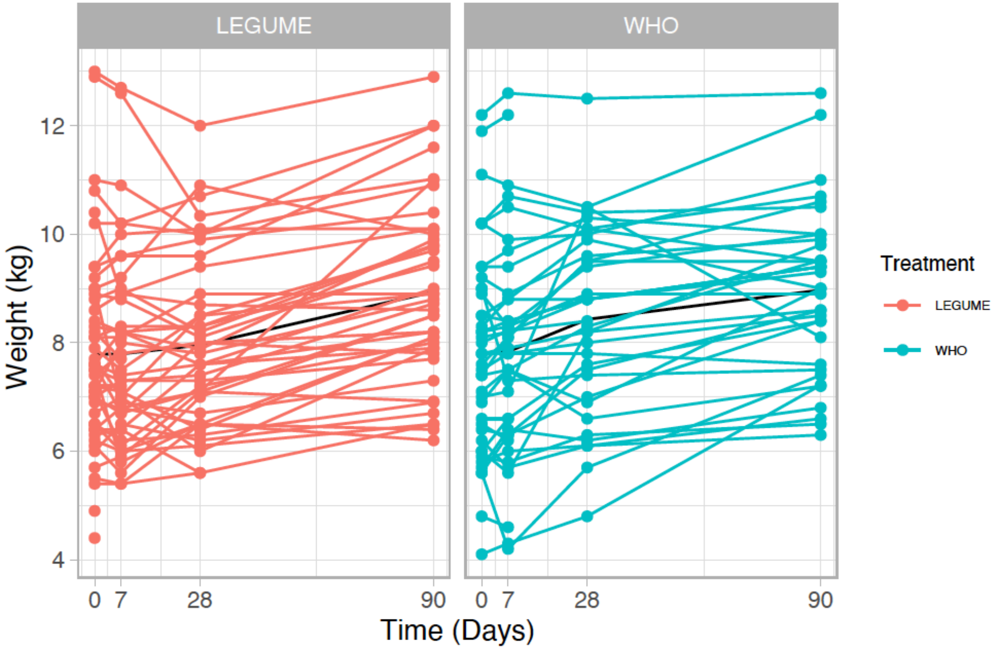

a) Represents individual weight between treatments across time (days) of children that presented with no oedema upon admission.
b) Represents individual weight between treatments across time (days) of children that presented with oedema upon admission.
Black lines represents the mean
